# Mesenchymal/Stromal Gene Expression Signature Relates to Basal-Like Breast Cancers, Identifies Bone Metastasis and Predicts Resistance to Therapies

**DOI:** 10.1371/journal.pone.0014131

**Published:** 2010-11-30

**Authors:** Cristina Marchini, Maura Montani, Georgia Konstantinidou, Rita Orrù, Silvia Mannucci, Giorgio Ramadori, Federico Gabrielli, Anna Baruzzi, Giorgio Berton, Flavia Merigo, Stefania Fin, Manuela Iezzi, Brigitte Bisaro, Andrea Sbarbati, Massimo Zerani, Mirco Galiè, Augusto Amici

**Affiliations:** 1 Genetic Immunization Laboratory, Department of Molecular, Cellular and Animal Biology, University of Camerino, Camerino, Italy; 2 Anatomy and Histology Section, Department of Morphological and Biomedical Sciences, University of Verona, Verona, Italy; 3 Surgical Pathology Section, Department of Oncology and Neuroscience, University of Chieti, Chieti, Italy; 4 Molecular Biotechnology Center, University of Torino, Torino, Italy; Massachusetts General Hospital, United States of America

## Abstract

**Background:**

Mounting clinical and experimental evidence suggests that the shift of carcinomas towards a mesenchymal phenotype is a common paradigm for both resistance to therapy and tumor recurrence. However, the mesenchymalization of carcinomas has not yet entered clinical practice as a crucial diagnostic paradigm.

**Methodology/Principal Findings:**

By integrating *in silico* and *in vitro* studies with our epithelial and mesenchymal tumor models, we compare herein crucial molecular pathways of previously described carcinoma-derived mesenchymal tumor cells (A17) with that of both carcinomas and other mesenchymal phenotypes, such as mesenchymal stem cells (MSCs), breast stroma, and various types of sarcomas. We identified three mesenchymal/stromal-signatures which A17 cells shares with MSCs and breast stroma. By using a recently developed computational approach with publicly available microarray data, we show that these signatures: 1) significantly relates to basal-like breast cancer subtypes; 2) significantly relates to bone metastasis; 3) are up-regulated after hormonal treatment; 4) predict resistance to neoadjuvant therapies.

**Conclusions/Significance:**

Our results demonstrate that mesenchymalization is an intrinsic property of the most aggressive tumors and it relates to therapy resistance as well as bone metastasis.

## Introduction

Despite progress in both knowledge and treatment, breast cancer remains the major cause of morbidity and mortality in Western Countries [1]. Mounting clinical and experimental evidence suggests that the shift of carcinomas towards a mesenchymal phenotype is a common paradigm of both resistance to therapy and tumor recurrence. Pharmacological and radiotherapeutic treatments induce the acquisition of mesenchymal features and increased cell motility [2]–[7]. In HER-2/neu experimental tumors, the anti-apoptotic mutations induce an aberrant evolution of the stroma [8]. The spontaneous development of mesenchymal tumors after epithelial cell regression has been proposed as a model of tumor recurrence [9]. This evidence demonstrates that the capacity to generate mesenchymal tumor cells is inherent in carcinomas, and suggests they could spontaneously evolve into mesenchymal tumors if the epithelium is attacked.

Despite increasing awareness of the contribution of mesenchymal-like cells to cancer progression, the real incidence of mesenchymalization in human carcinomas still remains elusive and has not yet entered clinical practice as a crucial diagnostic paradigm. Furthermore, in the past few years, emerging evidence that mesenchymal stem cells (MSCs) may support [10]–[12] and generate tumors [13]–[16], as well as the recent discovery that cancer stem cells exhibit a mesenchymal phenotype [17], suggests that carcinoma mesenchymalization is a multi-faced phenomenon which may also proceed in ways alternative to epithelial-to-mesenchymal transition. Taken as a whole, these findings highlight the importance of relating the occurrence of this phenomenon to disease outcome in clinic settings.

From a mammary carcinoma spontaneously developed in HER-2/neu transgenic mice, we previously established a carcinoma derived mesenchymal tumor cell lineage, called A17 [18], capable of developing highly aggressive mesenchymal tumors when injected into syngeneic mice. Here, we describe three mesenchymal/stromal-signatures which A17 cells share with mesenchymal stem cells (MSCs) and breast stroma, and show that these signatures: 1) significantly relates to basal-like breast cancer subtypes; 2) significantly relates to bone metastasis; 2) are up-regulated after hormonal treatment; 3) predict resistance to neoadjuvant therapies.

## Results

### CaMTCs exhibit different signaling pathways compared to epithelial cells

**Figure 1:**
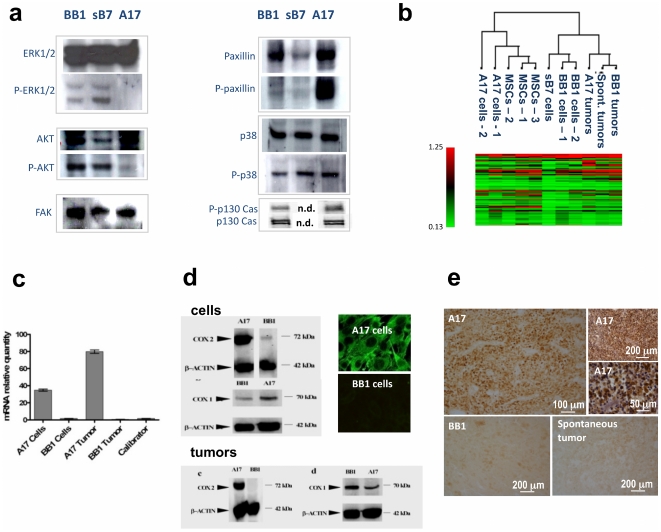
Signal transduction-related profile of A17. A17 cells exhibit a clearly different pattern of expression and activation (phosphorylation) of key signal transduction molecules compared to syngeneic epithelial cell lines (BB1 and sB7) (**a**). ERK1/2 or Akt proved to be constitutively expressed but not phosphorylated in A17 cells. In contrast, P-ERK1/2 and P-Akt were found in both BB1 and sB7 cells, where they are presumably recruited downstream of the HER-2/neu signaling pathway. Furthermore, whereas both epithelial and A17 cells proved to express Focal Adhesion Kinase (FAK), we found an increased phosphorylation of paxillin and p130 Cas in A17 cells, which is in line with the constitutively motile phenotype of these mesenchymal cells. p-38 was expressed and activated in both epithelial and A17 cells. Microarray analysis restricted to 96 signal transduction-related genes show the A17 cell profile to be more related to that of MSCs than that of syngenic epithelial cells (BB1). However, the A17 tumor profile was shown to be more correlated to that of epithelial tumors than that of epithelial or mesenchymal cells (**b**). The differential expression of *COX-2* in A17 compared to epithelial cells was confirmed at the transcriptional level through quantitative real-time PCR (**Figure 1c**). Western Blot analysis confirmed the differential expression of COX-2, but not of COX-1, in A17 cells and tumors comapared to BB1 cells and tumors (**figure 1d**). Differential expression of COX-2 protein between A17 and BB1 was also confirmed by immunocytochemistry on cell cultures (Figure 1d, right-up panles) and immunohistochemistry on tumor slices (**figure 1e**).

First, we characterized A17 cells for the expression and activation state of the MAP kinase ERK1/2, p-38, and the Ser/Thr Kinase Akt, which are known to be key molecules in cancer cell signaling pathways. Interestingly, we found that A17 cells exhibited a distinctive pattern of expression and activation of these molecules with respect to syngeneic epithelial cancer cells (BB1 and sB7) (**Figure 1a**).

We previously showed that A17 exhibited a stemness-related gene signature which was virtually identical to that of MSCs and an angiogeneic-related signature which had significantly higher correlation to that of MSCs with respect to that of syngeneic breast cancer cells (BB1) [10]. Herein our comparative analysis of A17s, MSCs and BB1s was extended to include a set of 96 genes involved in the most important cancer signaling pathways (**Table S1**). In accordance with previously described transcriptional analysis, A17 cells proved to be more highly related to MSCs than to epithelial lineages (BB1 cells and tumors, sB7 cells, Spontaneous tumors) also for signal transduction-related genes (**Figure 1b** **and Table S2**). However, A17 tumors proved to be more closely correlated to the epithelial tumor profile (BB1 and Spontaneous tumors) than to that of mesenchymal or epithelial cells.

Several cancer genes showed different expressions in A17 cells with respect to syngeneic epithelial cells (BB1 and sB7). Differences in angiogenesis-related genes have been shown previously [10]. Among the cancer signal transduction pathway-related genes (**Table S1**), *dual specificity phosphatase 1 (Dusp1)* and *COX-2* (also known as *PTGS2*) were significantly over-expressed (2.34-fold, Q value = 0.008 and 3.77-fold, Q value = 0.029, respectively) in the mesenchymal profile (A17 cells, A17 tumors, MSCs) with respect to all the epithelial lineages.

### COX-2 is a mesenchymal hallmark in tumors

**Figure 2:**
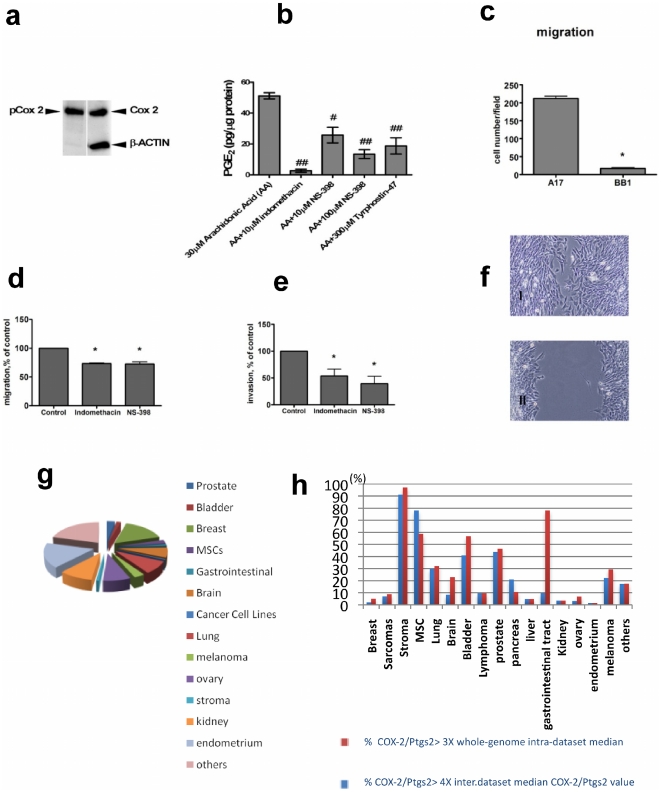
COX-2 was enzimatically active in A17 cells and regulated motile phenotype. Expression of the active (phosphorylated) isoform of COX-2/PTGS2 was confirmed by western blotting in not-treated A17 cells (**a**). PGE_2_ production (**b**) was assessed on A17 cells treated with Arachidonic Acid (control condition), preincubated with indomethacin (10 µM), NS-398 (10 µM and 100 µM) or Tyrphostin 47 (300 µM) for 30 minutes. Bars represent mean ± SD of three independent experiments with triplicate samples. # # p<0.01; # p<0.05 in a standard Student *t*-test. A17, but not BB1, exhibited an efficient motility throughout the Boyden chamber (**c**). Blocking COX-2/Ptgs2 with both selective (Indometacin) and not-selective (NS-398) inhibitors significantly reduced the motility of A17 cells in Boyden chamber assay (**d**) as well as A17 invasivity in matrigel (**e**). Bars represent mean ± SEM of three independent experiments with triplicate samples. * p<0.01 in a standard Student *t*-test. Treatment with Indometacin also inhibited A17 cell migration, within 18 h, into the gap after scraping of the confluent sheet (f, I:control; II, indometacin treated). Representative data from three independent experiments are shown. An in silico analysis on 2789 samples including varied types of human tumors, MSCs, and breast stroma (g) demonstrated the highest levels of COX-2 expression in breast stroma and MSCs (**h and Table S4 online**).

The importance of *COX-2* in growth, vasculogenesis and invasiveness has been widely documented in various types of carcinoma, both in clinical and experimental studies. However, our microarray analysis suggests that *COX-2* is a key molecule in the malignant phenotype of mesenchymal tumor cells. In order to investigate this hypothesis, the expression and functional activity of *COX-2* were further investigated. We confirmed at transcriptional and protein level the overexpression of COX-2 in A17 compared to syngeneic epithelial lineages (**Figure 1c–e**). Furthermore, we also observed that COX-2/PTGS2 expressed by A17 was enzymatically active, as it was phosphorylated (**Figure 2a**) and the PGE2 production by A17 cells proved to be sensitive to both non-selective (Indometacin) and selective (NS-398 and Tyrphostin) COX-2/PTGS2 inhibitors (**Figure 2b**). Moreover, we observed that the differential expression of COX-2/PTGS2 between A17 and BB1 cells was epigenetically regulated (**Figure S1**). Finally we found that COX-2/PTGS-2 was implicated in promoting cell motility and invasiveness of A17, given that its blocking by means of selective or non-selective inhibitors significantly hampered A17 migration through Matrigel and motility in vitro (**Figure 2e and f**).

Afterward, we were interested in mapping *COX-2* expression in human tumors. To this end, we analyzed *COX-2* expression values in a cohort of 2789 microarray datasets of human breast stroma, MSCs, and varied tumor histotypes, collected from publicly available databases (**Figure 2g** **and Table S3**). In order to minimize false discovery due to normalization errors, we cross-validated the data by two complementary approaches. First, we calculated the percentage fraction of samples expressing *COX-2* values higher than three times the whole-genome intra-sample median value. This approach is not affected by the inter-sample noise due to normalization error and captures information about whether *COX-2* is both a highly expressed gene within the transcriptome of a given sample. Secondly, we calculated the percentage fraction of samples where *COX-2* expression values were higher than three times the *COX-2* median value across all samples of the dataset. Both approaches demonstrated that stroma samples expressed the highest values of *COX-2*, closely followed by MSCs and then bladder and prostate cancers, demonstrating that COX-2/PTGS2 is a hallmark of the mesenchymal phenotype (**Figure 2h** **and Table S4**). Gastrointestinal stromal tumors exhibited very high *COX-2* levels using the first approach, but it was not confirmed in the second one

### A17 stemness-, angiogenesis- and signal transduction-signatures are over-represented in MSCs and Breast Stroma

**Figure 3:**
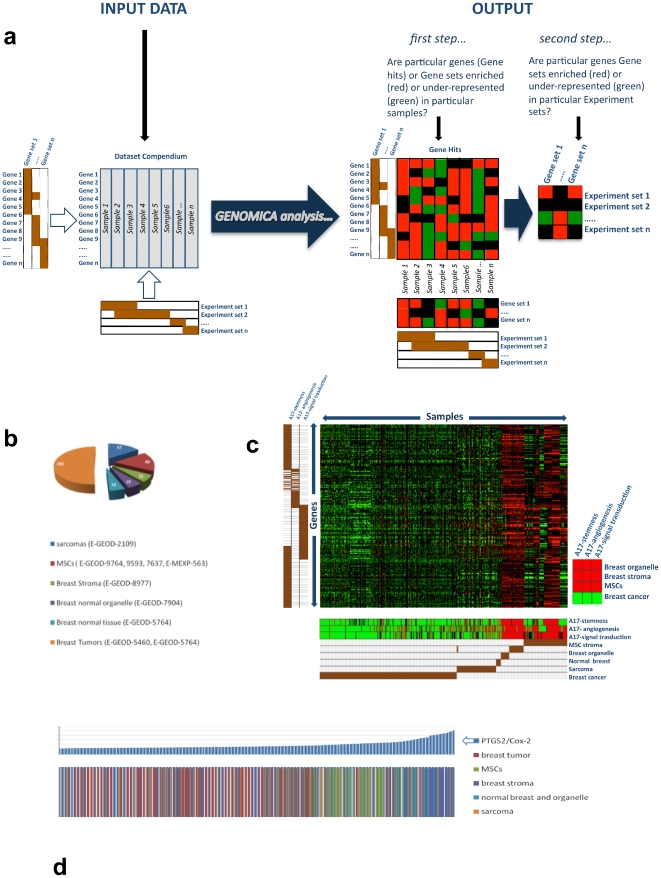
A17-signatures are enriched in MSCs and breast stroma. The GENOMICA algorithm makes it possible to quantitatively and statistically evaluate the enrichment of genes of interest in the samples of a given compendium of microarray datasets (**a**). Input data consist of the Dataset compendium and of a collection of Gene sets, the enrichment of which has to be tested. Optionally, samples may be grouped in Experiment sets. In the example in the Figure, input data comprise a Dataset Compendium with *n* samples, *n* Gene Sets and *n* Experiment sets; Gene set 1 includes genes 1 to 6, whereas Experiment set 2 includes samples 2 to 6, etc. Genes are scored as over- or under-expressed if their values are higher or lower than given cut off values established by the researcher. GENOMICA analysis consists of statistical evaluation, according to the Hypergeometric distribution, of whether the fraction of over-expressed (or under-expressed) genes in each sample includes a higher-than-randomly-expected fraction of genes from a particular gene set. If samples have been grouped in Experiment sets, a second step of analysis assesses whether specific gene sets are enriched in particular Experiment sets. Output consists of three heat maps: the first (Gene hits) depicts over-expressed (red squares) or under-expressed (green squares) genes in each *sample*; the second (beneath the Gene hits) depicts enriched (red squares) or under-represented (green squares) gene sets in each *sample*; the third (on the right of Gene Hits) depicts whether particular *Gene sets* are enriched in particular *Experiment sets*. Heat maps report only genes (or Gene sets) which met the criteria of statistical significance in at least 1 sample (or Experiment set). Black squares indicate genes (or Gene sets) which did not meet the criteria of statistical significance. GENOMICA analysis on a cohort of 360 publicly available human microarray datasets (**b**) demonstrated that all three A17 signatures are significantly enriched (red squares) in breast organelle, breast stroma and MSCs samples, but are significantly under-represented (green squares) in breast cancers (**c**). Plotting samples along an increasing order of the *COX-2* expression value, it turned out that MSCs and Breast stroma expressed the highest values of *COX-2* (**d**). The upper histogram displays increasing values of COX-2/PTGS2. The bars beneath depict samples ordered according to their COX-2/PTGS2 expression value. The different colors indicate the respective sample category each sample belongs to (breast cancer, MSCs, etc.).

We were interested in systematically relating carcinoma-derived mesenchymal tumor cells to varied human breast cancer histotypes and their potential clinical outcome. To this end, we took advantage of a recently developed computational algorithm, named GENOMICA [19], which makes it possible to quantify and statistically evaluate the enrichment of one or more gene sets in all samples of a given microarray dataset compendium (**Figure 3a**). Specifically, it tests whether the fraction of over- or under-expressed genes in each profiled sample includes a higher than randomly expected fraction of genes from one or more gene sets under analysis. Cut-offs for over- and under-expression are established by the user as a parameter of the analysis. A second step in the analysis assesses whether particular sample groups (experiment sets such as MSCs or Basal-like tumors, etc.) preferentially over- or under-express particular gene sets. In both steps, statistical evaluation of the enrichment is inherent in the computational algorithm. It calculates the p value of the fraction of over- or under-expressed genes according to hypergeometric distribution.

First, we compiled 3 gene sets, thereafter referred to “A17 signatures”, (**Table S5**) as follows: 1) **A17-stemness signature**: comprised genes of a stemness-related gene microarray the expression of which in A17 cells was higher or equal to the intra-sample median value; 2) **A17-angiogenesis signature**: comprised genes of an angiogenesis-related gene microarray the expression of which in A17 cells was higher or equal to the intra-sample median value; 3) **A17-signal transduction signature**: comprised genes of a cancer signal transduction-related gene microarray the expression of which in A17 cells were higher or equal to the intra-sample median value.

In order to relate the A17 phenotype with that of other mesenchymal phenotypes involved in tumorigenesis, we analyzed the enrichment of these A17 Signatures in a compendium of 360 publicly available human whole-genome microarray datasets, including samples of MSCs, breast stroma, breast cancer and varied types of sarcoma (**Figure 3b**).

After concatenating datasets, we normalized the entire compendium with the Robust Multiarray Average (RMA)-algorithm. The dataset samples included in the compendium were grouped into 6 Experiment Sets as follows: 1) Breast Cancer: comprised whole-genome microarray datasets of breast tumors; 2) Normal Breast: comprised whole-genome microarray datasets of normal tissue from invasive ductal or lobular breast carcinomas. 3) Breast Stroma: comprised whole-genome microarray datasets of stroma samples from normal or tumor breasts; 4) Breast Organelle: comprised whole-genome microarray datasets of breast organelles; 5) MSCs: comprised whole-genome microarray datasets of MSCs; 6) Sarcomas: comprised whole-genome microarray datasets of varied types of sarcomas.

All three gene sets were significantly over-represented in MSCs and breast stroma and significantly under-represented in breast cancers (**Figure 3c**). Sarcomas did not exhibit significant over-expression or under-expression of any of A17-signatures. Accordingly, breast stroma samples, MSCs and breast organelle expressed the highest values of *COX-2* (**Figure 3d**). However, both A17-signatures and *COX-2* were heterogeneously expressed in breast cancers, indicating subsets of breast cancer that over-express a mesenchymal related phenotype.

### A17-signatures identify ER-negative breast cancers

In order to systematically relate breast cancer mesenchymalization to any clincopathologic parameters, we analyzed the A17-signature expression using two publicly available microarray datasets obtained from two independent studies carried out on human patients. The former [20] comprised 295 human breast cancers with mixed ER, grade and lymph node status, the latter [21] comprised 286 human breast cancers, which were all lymph node negative.

**Figure 4:**
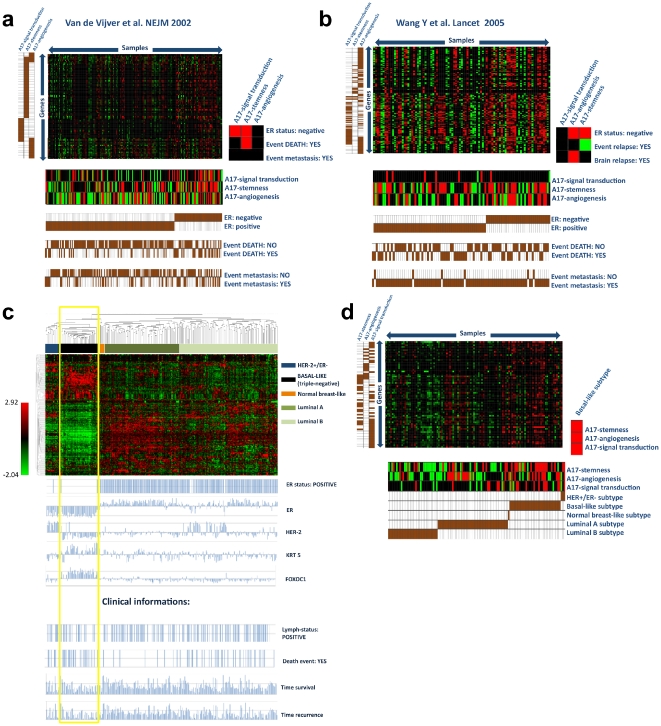
A17-signatures are enriched in basal-like breast tumors. Module map analysis of Van the Vijver's (**a**) and Wang's (**b**) cohort indicated that A17-signatures were enriched in ER-negative human breast cancers. ‘Intrinsic subtypes’ of breast cancers with different clinical outcomes were identified on the Van de Vijver's cohort based on the clusterization of the most differentially expressed genes (**c**). Module map analysis using these subtypes as experimental groups demonstrated that all A17 signatures were significantly enriched in the basal-like subtype (**d**).

In Van de Vijver's cohort (**figure 4a**), A17-stemness and signal transduction-signature were significantly enriched in ER-negative tumors, and the A17-stemness signature relates to the death of the patient. In Wang's cohort (**Figure 4b**), the A17-stemness signature was confirmed to be enriched in ER-negative tumors, but was inversely related to relapse events, suggesting a lower prognostic value of this signature in lymph node negative tumors. The A17-signal transduction signature was not significantly enriched in ER-negative tumors. On the contrary, the A17-angiogenesis signature was over-expressed in ER-negative tumors and was related to brain relapse. These data suggested that A17-signatures were enriched in ER-tumors, with some differences related to the lymph node status.

### A17-signatures relate to basal-like breast tumors

Based on the most differentially expressed genes, Sorlie et al. [22] previously identified five ‘intrinsic subtypes’ of breast cancer, strongly related to different clinical outcomes: normal-like, luminal type A, luminal type B, HER-2-like and basal-like. Tumors referred to as basal-like, or triple-negative tumors, exhibit the worst prognosis and are recognized by the combination of ER-/PR-/HER-2- negativity and KRT5-/KRT14-/FOXC1-/FABP4- positivity. In accordance with that method, unsupervised clusterization of Van de Vijver's dataset filtered by a standard deviation of ≥0.4 allowed us to identify the same five breast cancer subtypes described by Sorlie et al. As expected, the basal-like tumors showed a higher frequency of patient death, reduced time recurrence and survival (**Figure 4c** **, yellow frame**).

Module map analysis of the Van de Vijver's cohort showed that basal-like tumors were significantly enriched in all three A17-signature (**Figure 4d**).

### A17-signatures are over-expressed in bone metastasis compared to brain or lung metastasis

**Figure 5:**
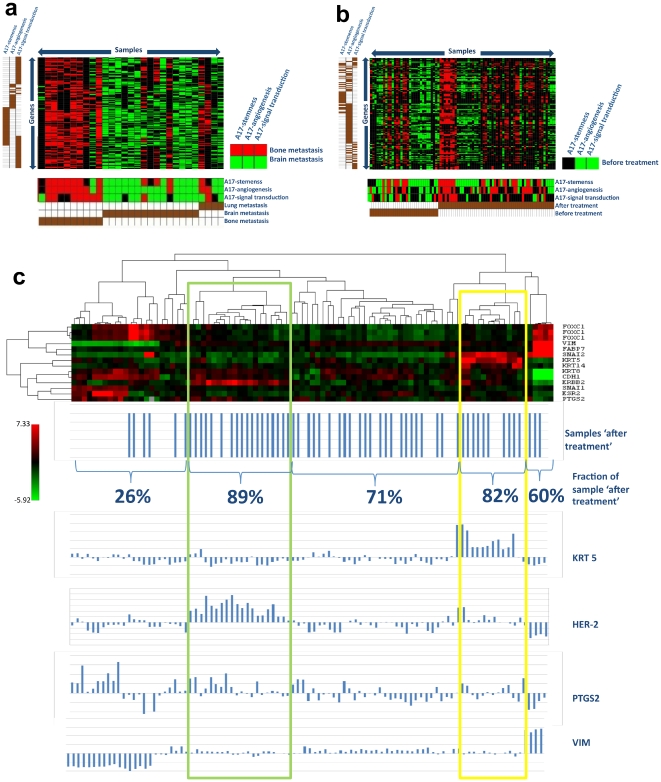
A17-signatures are enriched in bone metastasis and are up-regulated after hormonal therapy in ER-positive tumors. Module map analysis of public available microarray dataset of human metastatic tumors disseminated from primary breast cancers demonstrated that A17-signatures were significantly over-represented in bone metastasis samples and under-expressed in brain metastasis samples (**a**). Module map analysis on murine xenotransplats of breast tumor cells before and after hormonal therapy showed that A17-signatures were significantly under-represented in samples before treatment (**b**). Hierarchical clusterization for a restricted gene set of epithelial, basal-like and mesenchymal markers (**c**) showed that the highest percentages of ‘after treatment’ samples fell in two clusters, which were characterized by the higher-then-median expression of HER-2 and KRT5, respectively.

Cancer cells need to acquire a mesenchymal phenotype to disseminate and form metastasis, but it is commonly thought that metastatic cells revert to an epithelial phenotype after reaching a permissive distant site. However, as the most aggressive breast cancers may stably exhibit a mesenchymal phenotype, it is conceivable that distant metastasis might also relate to the mesenchymal phenotype. In order to interrogate this hypothesis, we analyzed the A17-signature enrichment in a restricted cohort of 29 human distant metastases (Arrayexpress, accession number E-GEOD-14017) disseminated from primary breast cancers to lung, brain or bone. Interestingly we found that all three A17-signatures were significantly over-expressed in bone metastasis, but significantly under-expressed in brain metastasis (**Figure 5a**). Lung metastasis did not exhibit significant over- or under-expression for these signatures.

### A17-signatures are over-expressed after hormonal therapy

ER-positive tumors, with or without HER-2 over-expression, usually undergo hormonal therapy. The most used therapeutic protocol is based on the use of tamoxifen, but recently alternative drugs have been developed. Although it is accepted that these treatments frequently improve clinical outcome, a large percentage of tumors are ultimately resistant.

In order to assess whether therapy resistance might underlie the change of the tumor phenotype toward a mesenchymal/basal phenotype, we collected a cohort of 93 microarray datasets comprising murine xenografts of human tumors before and after hormonal therapies. The cohort was obtained by concatenating microarray data from five independent studies (**Table S6**). After concatenating datasets, the cohort was normalized by the RMA-algorithm and underwent module map analysis to search for the over- or under-expression of A17-signatures.

Principally, the cohort included murine xenografts of human ER-positive breast cancer cells (MCF-7), with or without HER-2 over-expression, supplemented or not with estrogen (E2). A limited number of samples of human colon carcinoma (COLO-205) or melanoma cells (SK-MEL5) were also included. Colon carcinoma and melanoma xenografts underwent PEP008 treatment and some samples of MCF-7 underwent PEP008-based treatment, but most of the MCF-7 xenografts were treated with a tamoxifen-based therapy. We assigned samples to two experiment sets, which were “before” and “after treatment.”

As expected, module map analysis showed that A17-signatures (angiogenesis and signal transduction) were significantly under-expressed in “before treatment” groups of samples (**figure 5b**). This result indicates that post-treatment samples have a higher expression of A17-signatures compared to pre-treatment samples.

In order to further characterize the phenotypic change induced by the treatment, we clusterized the expression values of a restricted gene set including well known mesenchymal, basal-like and epithelial markers (**Figure 5c**). Interestingly, we found that the highest fractional percentages of ‘after-treatment’ samples fell into two clusters, which were identified by the highest expression of HER-2 and KRT5, respectively.

### A17-signatures predict resistance to neoadjuvant therapies

Farmer et al [23] recently identified a stroma-related signature which predicts resistance to neoadjuvant therapies in human breast cancer, but fails to function as an intrinsic prognostic marker of clinical outcome. The results shown above demonstrated that the combination of our A17 signatures are intrinsically effective in identifying the most aggressive breast tumors (basal-like) and bone metastasis, in predicting clinical outcome and in characterizing changes associated to hormonal therapy-resistance in ER-positive breast tumors. In order to assess whether A17-signatures might also be effective in predicting resistance to neoadjuvant therapies, we mapped their enrichment in a cohort of human samples obtained by concatenating Farmer's study (102 samples, all ER-negative) with a previous study from the same laboratory [24] (125 samples, all ER-negative). Both of the two independent studies included human breast tumors subjected to neoadjuvant non-taxane regimens with 5-fluorouracil, epirubicin, and cyclophosphamide (FEC). In accordance with the original papers, samples were assigned to two types of outcome, which were complete or not complete pathological responses (pCR and npCR, respectively). pCR was defined as the disappearance of the invasive component of the primary tumor after treatment, with at most a few scattered tumor cells detected by the pathologist in the resection specimens.

As the original studies were based on a similar experimental design, the datasets were normalized as described by Segal et al. [19]. Briefly, the original datasets were log2 transformed and normalized separately by subtracting the mean value of the genes across the samples from each data point. After normalization, the original datasets were concatenated in an unique dataset.

**Figure 6:**
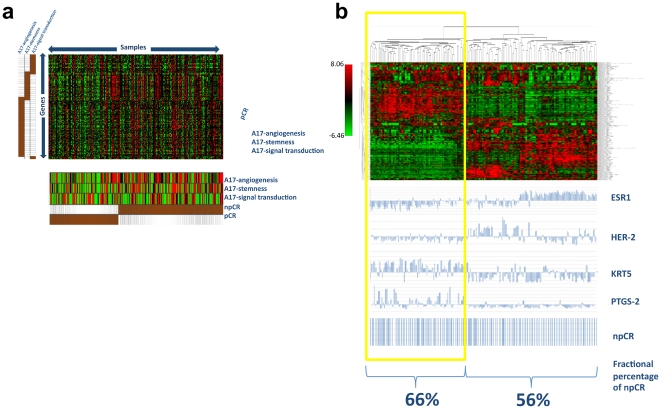
A17 signature under-expression predicts response to neoadjuvant therapy. Module map analysis on a cohort of samples completely (pCR) or not completely (npCR) sensitive to neoadjuvant therapy demonstrated that A17 signatures are under-expressed in pCR samples (**a**). Hierarchical clusterization of the dataset filtered for the most differentially expressed genes (SD>1.5) generated two major clusters, one of which exhibited features of Basal-like tumors (**b**: yellow frame). The basal-like cluster included a higher percentage of npCR samples than the other cluster.

Interestingly, pCR proved to significantly under-express A17-stemness and A17-signal transduction signatures, whereas npCR did not (**Figure 6a**).

Unsupervised hierarchical cluster analysis of the most differentially expressed genes (SD>1.5; 313 genes passed filter) of the entire cohort confirmed npCR samples to be enriched in the subtype with a basal-like phenotype (ER-negative, HER-2-negative, KRT5-positive) compared to the ER-negative/HER-2-positive-subtype (**Figure 6b**). Interestingly, COX-2 were over-expressed in this basal-like subtype, confirming this molecule as a marker of basal-like breast tumors.

### Basal-like breast tumor subtypes are breast cancers

Our results demonstrate that the most aggressive (basal-like subtype), or therapy-resistant breast tumors, frequently over-express mesenchymal/stromal signature. However, that does not necessarily means they are mesenchymal tumors. Thus, we were interested in comparing the general expression analysis of all breast cancer subtypes to that of tumor-related mesenchymal phenotypes. To this end, we systematically analyzed the above-described RMA-normalized human cohort (**Figure 3a**), including breast cancers, along with MSCs, breast stroma and sarcoma samples (**Figure S2 and S3**). Taken as a whole, our analysis shown that, although significantly enriched for mesenchymal/stromal genes, basal-like tumors prevalently exhibit an epithelial signature.

## Discussion

Breast cancer is a multi-faced disease where histologically divergent cell populations interact to carry out aberrant programs of development. All therapeutic approaches that have been developed in the last few decades suffer from limited efficacy, due to the wide heterogeneity of tumors and to their ability to elude treatment by activating alternative developmental programs. Discovering biological paradigms that could characterize and identify the “heart” of the most aggressive tumors beyond their apparent heterogeneity represents a crucial challenge for diagnosing tumor subtypes, forecasting its potential outcome and developing appropriate therapies.

Different lines of evidence converged toward the notion that breast tumors have a mesenchymal “heart.” However, for decades the major efforts in the fight against breast cancer have searched for diagnostic parameters and therapeutic targets directly or indirectly related to the epithelial phenotype, such as HER-2, estrogen receptors, progesterone receptors, anatomical architecture of the gland, and ductal or luminal morphology. Only in the last years, the convergence of several studies from different areas of research has given rise to the notion that mesenchymalization is an intrinsic potential of breast cancers, and that it can proceed from alternative mechanisms other than EMT, such as the neoplastic transformation of mesenchymal or epithelial stem cell precursors [2]–[9], [11], [13]–[16], [25], [26].

The key finding of our study is that the shift toward a mesenchymal/stromal phenotype is an intrinsic property of most aggressive (basal-like) and therapy-resistant primary tumors, as well as of bone metastasis. Although our A17 signatures have been identified in a murine model of mammary cancer, they strongly related to human MSCs and stromal phenotype and proved to be unexpectedly effective in identifying basal-like subtypes, bone metastasis and therapy reistant tumors in humans. This suggest how mesenchymalization in breast carcinomas may have a cross-species impact.

A mesenchymal phenotype of basal-like tumors has been recently shown by Sarriò et al [27], which revealed how these aggressive subtypes of tumors frequently exhibit over-expression of some EMT markers (vimentin, alpha-SMA, SPARC) and cadherin switching (down-regulation of E-cadherin and up-regulation of N-, P- and cadherin-11). We provide herein additional data in support of this idea, showing that human basal-like tumors strongly over-express a panel of function-restricted gene sets (angiogenesis, signal transduction and stemness-related) that are highly specific to carcinoma-derived mesenchymal tumor cells, but also strongly related to breast stroma and mesenchymal stem cells (A17-signature).

The fact that unsupervised hierarchical clustering of both whole-genome and array-set restricted genes didn't directly clusterize basal-like tumors with any mesenchymal phenotype (breast stroma, mesenchymal stem cells, sarcomas) indicates that basal-like tumors only rarely acquire a completely sarcomatoid phenotype, whereas the appearance of mesenchymal traits are more frequently mixed with a variable fraction of epithelial features. In accordance with this notion, it has been previously shown that basal-like tumors, more frequently than other breast tumor types, exhibit the presence of restricted spindle cell tumor areas [28].

Interestingly, despite the fact that the A17-signature was enriched in ER-negative and even more basal-like primary tumors, its expression was up-regulated in ER-positive tumors after hormonal therapy. This suggests that therapeutic treatments specifically addressed to luminal cancer cells can prompt the shift toward a mesenchymal, basal-like phenotype. ER-negative tumors are preferentially subjected to preoperative (neoadjuvant) chemotherapy, which leads to the disappearance of the primary tumor (pathological complete response; pCR) in 20–30% of ER-negative tumors, whereas its efficacy is limited to less than 10% of ER-positive tumors [29]–[31]. Farmer et al [23] described a stroma-related gene signature capable of predicting resistance to neoadjuvant chemotherapy. However, this stroma-related signature was not able to capture information about the innate aggressiveness of the tumor, thus failing to forecast the clinical outcome of tumors independently of neoadjuvant treatment. Authors cited a previous study which described a new stroma-derived prognostic predictor (SDPP) obtained by microdissected tumor stroma [32]. They explained the different results in the two studies on the basis of the fact that their signature assessed only a single aspect of gene expression in the stroma, whereas SDPP was a composite signature that takes into account multiple cell types and processes occurring in the stroma. A17-signature proved, indeed, to be able to identify more aggressive and lethal tumors, as well as to predict resistance to neoadjuvant therapies in ER-negative tumors.

The key feature of the A17-signature is that, although strongly related to breast stroma and MSCs, it derives from carcinoma-derived mesenchymal tumor cells, thus identifying the neoplastic counterpart of the mesenchymal phenotype in breast cancers. This high neoplastic-mesenchymal specificity may explain the prognostic other than predictive efficacy of A17-signature.

The major challenge in the fight against tumors is to eradicate metastasis in distant sites. Bone is the site of metastatization for up to 70% of patients with advanced breast cancer [33]. The onset and growth of a metastatic focus in a distant site is due to intrinsic features of both the host microenvironment and the disseminating cancer cell. The most commonly accepted model of carcinoma metastatization provides that epithelial cells incur in EMT to actively disseminate from the primary tumor, but then revert toward an epithelial phenotype after reaching the permissive distant site. We show herein that bone metastasis retains a mesenchymal/stromal/basal-like phenotype. It cannot be known whether this phenotype is due to a selection process by the bone sites or to an intrinsic property of the disseminating cells capable of reaching the bones. However, the evidence of the mesenchymal nature of breast-cancer derived bone metastasis strongly suggests a new approach to searching for more targeted therapies.

A key feature of our A17-signature is the over-expression of COX-2. For a long time this molecule has been known to significantly correlate with poor prognosis in breast cancer. However, as far as we are aware, nobody has shown that its expression is restricted to breast stroma and MSCs and can identify breast cancer mesenchymalization. Our results propose COX-2 as an additional bio-marker of breast cancer mesenchymalization and target for therapy in addressing the most aggressive tumors, i.e. the basal-like ones.

## Methods

### Cells and tumors

A17 and BB1 cell lines were established from a mouse mammary carcinoma model (FVB/neuT transgenic mice) as previously described [18].

Mesenchymal stem cells (MSCs) were isolated from inguinal adipose tissues of FVB mice and identified on the basis of their immunophenotypical profile and their *in vitro* multilineage plasticity toward adipocytes, chondrocytes and osteocytes, as previously described^10^.

Experimental tumors were induced by s.c. injection of 1×10^5^ A17 or BB1 cells into the groins of 5–7 weeks-old female FVB/neuNT233 mice.

### Microarray

All the steps of microarray analysis for signal transduction-related genes were performed using reagents from Superarray Biosciences Corporation (Friederick, CA, USA), closely following the manufacturer's instructions. Briefly, total RNA was isolated from samples using the Qiagen RNeasy kit (Qiagen). The TrueLabeling-AMP™ Linear RNA Amplification Kit (SuperArray Bioscience Corp. Frederick MD) was used to amplify and label RNA for hybridization. Labeled cRNAs were hybridized overnight at 60°C with the arrays. After washing, hybridization was revealed by Chemioluminescence and the array image was captured by X-ray film and a flatbed desktop scanner. Data from arrays was analyzed using GEArray Expression Analysis Suite (Superarray). Quantitative expression values were corrected to the background, normalized with respect to the positive control genes included in the arrays, and reported as ratios to the mean values of normalization genes. We have deposited the raw data at GEO database under accession number GSE22663, we can confirm all details are MIAME compliant.

### Phospho-protein purification

10^7^ A17 cells were assayed with Phospho-Protein Purification kit (Qiagen, Milano, Italy) closely following manufacturer's instructions, with minor modifications.

### Real Time PCR

Quantitative PCR was performed on a Real Time PCR System (Stratagene, Mx3000P) using the real master mix kit (Eppendorf, Milano, Italy), containing SYBR Green (1mM dNTPs, 10mM (CH_3_COO)_2_Mg and 0.05U/µl HotMaster Taq polymerase). We used the following primers (400 nM) to amplify each target gene: COX-2/Ptgs2 (178 bp): 5′-TGCTCACGAAGGAACTCAGC-3′ for forward primer and 5′-CTCATACATTCCCCACGGTTTTG-3′ for reverse primer. GAPDH (97 bp), 5′-GAAGCTTGTCATCAACGGGAAG-3′ for forward primer and 5′-ACTCCACGACATACTCAGCAC-3′ for reverse primer. Reactions were carried out for 45 cycles following an initial template denaturation step of 90 s at 94°C. The cycle conditions were: 30 s at 94°C, 30 s at 57°C and 30 s at 72°C.

### Western Immunoblotting

Confluent cells were lysated directly into the 25 cm^2^ culture flasks using a boiling sample buffer without beta-mercaptoethanol (50 mM Tris pH 6.8, 2% SDS, 10% Glycerol). After electro-blotting onto polyvinylidene difluoride (PVDF) membranes (Immobilon P, Millipore, Milano, Italy) antigens were probed as follows: anti-COX-2/PTGS2 polyclonal rabbit primary antibody (Cayman, Ann Arbor, MI)(1∶1000), anti-COX-1 polyclonal rabbit primary antibody (Cayman, Ann Arbor, MI) (1∶200), Rabbit (polyclonal) Anti-Human FAK (Biosource International, cat.no. AHO0502) (1∶200), Anti-paxillin (Transduction Laboratories, Cat. No. P13520) (1∶1000), anti-Phospho-Paxillin (Tyr118) Antibody (Cell Signaling technology, Cat. No. 2541) (1∶750), anti-Akt Antibody (Cell Signaling Technology, Cat.no. 9272) (1∶500), anti-Phospho-AKT (Ser473) Antibody (Cell Signaling technology, Cat. no. 9271) (1∶1000), anti-p44/42 MAP Kinase (3A7) Mouse mAb (Cell Signaling technology, Cat. no. 9107) (1∶1000), anti-p44/42 MAP Kinase (Thr202/Tyr204) (E10) Monoclonal Antibody (Cell Signaling technology, Cat. no. 9106) (1∶1000), anti-P38 MAP Kinase Antibody (Biolabs, Cat. No. 9212) (1∶1000); anti-Phosphor-p38 MAP Kinase (Thr180/Tyr182) Antibody (Biolabs, Cat.no. 9211S) (1∶1000), anti-β-actin monoclonal primary antibody (Oncogene, Boston, MA) (1∶5000).

Monoclonal antibody (mAb) 1H9 to p130Cas was prepared in our laboratory, by immunizing mice with a recombinant protein encompassing amino acids 360–685 of mouse p130Cas cDNA sequence (a kind gift from Dr S. Hanks, Nashville, USA). 1H9 mAb specificity was tested by using, as a negative control, fibroblasts derived from p130Cas null mice (a kind gift from Dr H. Hirai and T. Nakamoto, Tokyo, Japan). 1H9 recognizes p130Cas by immunoprecipitation and western blotting and its reactivity was identical to that obtained with the p130Cas mAb from Transduction Laboratories (BD Biosciences Pharmingen, San Diego, CA, USA) (unpublished results). Phosphorylation of p130Cas was detected using the antibody pTyr-PY99 (Santa Cruz Biotechnology, Santa Cruz, CA).

The immunoreactive bands were detected with ECL detecting reagents (Amersham Bioscience, Little Chalfont, UK).

For p130 Cas immunodetection, cells were lysated with NP-40 lysis buffer (1% NP-40, 150mM NaCl, 50mM Tris pH 8) and p130 Cas was immunoprecipitated in G-Sepharose beads before western immunoblotting.

### Immunofluorescence analysis

A17 and BB1 cells grown to confluence on coverslips were fixed in ice-cold methanol at −20°C for 5 minutes, permeabilized in PBS containing 0.2% Triton X-100 for 7 minutes and blocked with PBS–10% BSA for 20 minutes. Cells were then incubated for 1 hour at 37°C with the polyclonal primary antibody against COX-2/Ptgs2 (Cell Signaling, Danvers, MA) diluted 1∶100 in a blocking buffer and, after washing, with the secondary antibody Alexa Fluor 488 goat anti-rabbit IgG (Molecular Probes, Eugene, Canada) diluted 1∶200. Coverslips were mounted on Mowiol (Sigma, St. Louis, MO) and the preparations were viewed using a Leica confocal TCS SP2 microscope.

### Immunohistochemistry

Animals were anesthetized with ether and perfused with 4% paraformaldehyde in 0.1 M phosphate buffer, pH 7.4. Tumors were excised from animals and further fixed by immersion in the same fixative, dehydrated and embedded in paraffin wax.

Paraffin sections of tumors were processed for immunohistochemistry using the avidin-biotin complex (ABC) technique. Briefly, deparaffinized sections were rehydrated and endogenous peroxidase activity was quenched by 15-min incubation in a solution of 3% hydrogen peroxide in methanol. After washing in 0.05 M Tris-HCl buffer (pH 7.6) sections were incubated with the anti-Ciclooxygenase-2 (1∶1000, Cayman Chemical, MI, USA ) overnight. After three washes, sections were then reacted with biotinylated swine anti-rabbit immunoglobulins (DAKO, Milan, Italy), diluted 1∶400, for 2 hours. The immunoreaction was detected using a Vectastain *Elite* ABC kit (Vector, Burlingame, CA, USA), and then visualized by 3,3 diaminobenzidine tetrahydrochloride (Dako, Milan, Italy) for 5–10 min. Finally, sections were dehydrated, coverslipped with Entellan, and observed in an Olympus BX51 photomicroscope equipped with a KY-F58 CCD camera (JVC). Control sections were processed as above, omitting the primary antibodies and no immunostaining was observed in these sections.

### COX activity

2×10^5^ cells were plated onto 24 well plates and grown in DMEM with 20% FBS for 2–3 days to reach confluency. Cells were washed twice with PBS and incubated with 30µM arachidonic acid in 1 ml of DMEM for 10 minutes at 37°C. To investigate the functional contribution of COX-2/Ptgs2 to total COX activity, cells were pretreated with the non-selective COX-1 and COX-2/Ptgs2 inhibitor indomethacin (10 µM) (Sigma, St. Louis, MO) or with COX-2/Ptgs2-selective inhibitor NS-398 (10 µM and 100 µM) (Sigma, St. Louis, MO) for 30 minutes. Cell proteins were detected using the Bio-Rad Protein Assay Dye Reagent (Bio-Rad, Hercules, CA). All experiments were conducted in triplicate.

To evaluate the effects of protein tyrosine phosphorylation on COX activity, cells were preincubated with the protein kinase inhibitor Tyrphostin 47 (300 µM) for 30 min.

### Prostaglandin E_2_ assay

Concentration of PGE_2_ in the cell incubation medium was determined by radioimmunoassay, using specific antibodies according to procedures reported elsewhere (Gobbetti *et al.*, 1999). The assay sensitivity was 7.5 pg/ml; intra- and inter-assay coefficients of variations were 8% and 12%, respectively. The PGE_2_ concentration was normalized to cell protein.

### Cell migration and invasion assays

Cell migration and invasion assays were performed using 24-well Transwell Boyden chambers (Corning Costar Corporation, Cambridge, MA).

Cell motility was measured by counting the number of cells crossing 8-micron pore size polycarbonate membranes coated with 10 µg/ml of fibronectin, and cell invasion was determined by counting the number of cells invading through a Matrigel-coated membrane that simulates basement membrane. Cells were harvested, washed twice with a serum-free medium, and re-suspended at 80,000 cells/ml. The upper chambers (inserts) containing uncoated or Matrigel-coated membrane were filled with 100 µl of cell suspension in DMEM containing 0.1% BSA, with or without the test substance, while the lower chambers (wells) were filled with 700 µl of the same cell-free media plus FBS (20%), that served as a chemo-attractant. Plates were placed in a humidified CO_2_ incubator for 24 h at 37°C. The inserts with membranes were then removed, the upper surface of the membrane was wiped with cotton swabs to remove non-migrated cells, and the membranes were fixed and stained with Diff-Quick staining solution (Dade-Behring Holding GmbH, Liederbach, Germany). Finally, the stained membranes were quickly washed with water, inverted and air-dried. Stained cells in three representative fields were counted at ×40 magnification. These assays were carried out in triplicate.

### Wound healing assay

The confluent A17 cell monolayer was wounded by scraping with a pipette tip, exposed to pharmacological treatments (10 µM indomethacin) and monitored for wound closure after 18 hours.

### Methylation-Specific PCR

DNA methylation patterns in the COX-2/Ptgs2 promoter were determined by Methylation-Specific PCR (MSP). Briefly, cells were cultured to subconfluence and harvested to extract genomic DNA using the QIAamp DNA blood mini kit (Qiagen, Germany). Genomic DNA was digested with a methylation-sensitive restriction enzyme HpaII, which could cleave a CpG site when it is not methylated. To ensure that the CpG site was not mutated and cleavable, the same DNA was digested with a methylation insensitive restriction enzyme MspI, which could cleave the CpG site irrespective of the methylation status. In short, when the CpG site was methylated, amplification of the HpaII digest should have produced a band similar to the one in the control batch (without any restriction enzyme). DNA (1 µg) was incubated at 37°C for 24 h in a 100 µl reaction for digestion containing 20 U of MspI, 20 U of HpaII (New England Biolabs Inc., Beverly, MA, USA), or no enzyme. PCR was carried out using Hot Master Taq DNA polymerase (5 Prime, Hamburg). Three sets of primers were designed to amplify the COX-2/PTGS2 promoter sequences containing one CpG site each: −569 nt, −465 nt, and −186 nt from the trascription starting site to produce 150, 130, and 170 bp amplicons, respectively. Primer sequences were: (150 bp) 5′-GTCCCTGGGAAAGGCGAGTG-3′ (sense) and 5′-GTTAATTTAATTTCTTCTAT-3′ (antisense); (130 bp) 5′-CTAATTCCACAAGTACAGAT-3′ (sense) and 5′-CCCCACTGGGGCGCAGTCTG-3′ (antisense); and (170 bp) 5′-AGAGGGCGGTGCAGCTCTCT-3′ (sense) and 5′-CTTTCCGCTTAGGCTTCCCC-3′ (antisense). After 2 min. of initial heat denaturing at 94°C, DNA was amplified by 40 cycles of denaturing at 94°C for 20 sec, annealing at 62°C for 20 sec and extension at 68°C for 20 s, followed by 5 min at 68°C.

### Treatment with 5-Aza-2′-deoxycytidine

BB1 cells were seeded at a density of 1×10^5^ cells/100 mm-dish and allowed to attach over 24 h. 5-Aza-2′-deoxycytidine (5-Aza-CdR) (Sigma) was added to the medium, at a final concentration of 1 or 10 µM and cells were cultured for 72 h. The medium was replaced every 24 h with a newly prepared medium containing 5-Aza-CdR. Under demethylated conditions, BB1 cells were incubated in the absence or presence of PMA 50 ng/ml for 4 h. Twenty four hours prior to stimulation, the medium was changed and cells were cultured in DMEM supplemented with 0.5% FBS. At the end of the treatment period, the genomic DNA was extracted for MSP analysis and the proteins were extracted for western analysis.

### GENOMICA analysis

Module mapping of comparative gene enrichment was performed using the Genomica Software, developed by Segal et al [19] and freely available on the web site: http://genomica.weizmann.ac.il.

Sources of the datasets and the normalization method are detailed in the result section for each analysis. In general, we chose the normalization method we thought to be the most appropriate for each compendium. Compendia the datasets of which belonged to the same original study were used as they were downloaded without any further normalization, except the mean gene centering of the genes. Compendia obtained by concatenating multiple datasets from independent studies with different experimental design were normalized according to RMA-algorithm and then gene mean centered. Compendia obtained by concatenating multiple datasets from independent studies, but with similar experimental design, were normalized separately before concatenating them, as described by Segal et al [19]. Briefly, expression values were Log2 transformed and processed by subtracting the mean value for each gene across all samples of the compendium from all data points, so that in all cases expression values of each data point were reported as positive or negative depending on whether it was higher or lower than the mean value of that gene across the samples.

Genes were scored as over-expressed or under-expressed if their value was respectively above or below cut-off values of fold-change respect to mean value. The cut-off we choose varied from ±1.3 to 2.0 depending on the compendium. Statistical analysis of the enrichment is an implicit function of the GENOMICA algorithm, which calculates P of the hypothesis that the fraction of over-expressed or underexpressed genes in particular samples was consistent with that randomly expected, according to the hypergeometric distribution. P<0.05 upon False Discovery Rate (FDR)-correction was accepted as statistically significant.

### Cluster analyses

Cluster analyses were performed using Cluster 3.0 software developed by Eisen et al. [34]. All analyses were carried out on normalized and log2 transformed dataset values. Uncentered Pearson-correlation was used as the similarity metrics and average linkage as the clustering method.

## Supporting Information

Table S1 Signal transduction-related microarray.
(0.16 MB PDF)

Table S2 Correlation indexes for the signal transduction-related gene profiles.
(0.04 MB PDF)

Table S3 Reference source of publicly available human microarray datasets.
(0.08 MB PDF)

Table S4 Percentage fraction of samples overexpressing Ptgs2.
(0.05 MB PDF)

Table S5 A17 signatures.
(0.26 MB PDF)

Table S6 Reference source of microarray dataset of samples undergone hormonal therapy.
(0.05 MB PDF)

Figure S1 DNA Methylation pattern in the COX-2/PTGS2 promoter were determined by Methylation-Specific PCR (MSP). Treatment with IL-1β, HGF and TGFβ for 2, 4 and 6 hours, was not able to induce the COX-2 expression in BB1, while resulted in increased levels of COX2 in the A17 cell (a). Three primers were designed for amplifying the sequence containing three side CpG in the promoter of COX2: −465 nt (A), −569 nt (B) and −186 nt (C) (b and c). COX2 promoter resulted hypermethylated in BB1 cells, but it was demethylated in A17 cells (d). The state of demethylation of the promoter COX2 was examined by MSP after treatment of cells BB1 for 72 hours with the substance demethylating 5-aza-CdR at a concentration of 1 and 10 µM (e).
(1.70 MB TIF)

Figure S2 Preliminary unsupervised cluster analysis of the most differentially expressed genes (SD>1.8), restricted to breast cancers only, confirmed that the dataset previously described in figure 3 comprises all five subtypes, including the basal-like one (ER-negative, HER-2-negative, KRT5-positive) (yellow square).
(8.20 MB TIF)

Figure S3 When breast tumors were clusterized together with mesenchymal samples (breast stroma, MSCs, sarcomas), basal-like samples clusterized with breast cancers, whereas breast stroma, MSCs and sarcomas formed distinct clusters (a). Thus, we supervised whole-genome clusters searching for any gene cluster that might relate basal-like to epithelial or mesenchymal samples. We identified three gene sets with an apparently interesting pattern of expression, and performed hierarchical clusterization of these genes separately (b). Cluster 1 comprised apparently peculiar genes of basal-like cells and normal breast/organelle samples. As expected, it included KRT5, KRT14 and KRT15. Hierarchical clusterization of these genes did not clearly separate epithelial and mesenchymal phenotypes, thus indicating that these genes failed to directly relate basal-like cells to non-basal breast cancer or mesenchymal phenotypes. Cluster 2 relates basal-like samples to non-basal breast cancers and breast stroma, whereas MSCs and Sarcomas formed distinct clusters. In Cluster 3, most of the basal-like samples fell into a large cluster which included all mesenchymal samples, along with a limited number of non-basal breast cancers. Finally we clusterized breast cancers and mesenchymal samples for the genes comprised in the stemness-, angiogenesis- and signal transduction-related arrays described above, both considering all the genes of the array or restricting analysis to those over-expressed by A17 cells (A17-signatures) (c). In all cases, basal-like samples clusterized more closely to breast than mesenchymal samples.
(9.18 MB TIF)
