# Supplementary material for: Mesenchymal/Stromal Gene Expression Signature Relates to Basal-Like Breast Cancers, Identifies Bone Metastasis and Predicts Resistance to Therapies
Source: PLoS One. 2010 Nov 30;5(11):e14131. doi: 10.1371/journal.pone.0014131 (PMC2994727; doi:10.1371/journal.pone.0014131)
Supplement: Table S1 — Signal transduction-related microarray. (0.16 MB PDF) [file pone.0014131.s001.pdf]

Position	Unigene	GeneBank	Description	Gene Symbol	A17 cell-1	BB1 cell-1	sB7 cell	A17 cell-2	BB1 cell-2	Spontaneous tumor	MSCs-1	MSCs-2	MSCs-3	A17 tumors	BB1 tumors
1	Mm.207354	NM_011076	ATP-binding cassette, sub-family B (MDR/TAP), member 1A	Abcb1a	0.16	0.21	0.21	0.14	0.2	0.2	0.21	0.18	0.21	0.19	0.28
2	Mm.146649	NM_011075	ATP-binding cassette, sub-family B (MDR/TAP), member 1B	Abcb1b	0.17	0.14	0.22	0.13	0.13	0.1	0.19	0.17	0.15	0.15	0.15
3	Mm.6645	NM_009652	Thymoma viral proto-oncogene 1	Akt1	0.22	0.21	0.27	0.2	0.36	0.14	0.38	0.31	0.39	0.23	0.24
4	Mm.209903	NM_009715	Activating transcription factor 2	Atf2	0.24	0.18	0.21	0.32	0.33	0.24	0.42	0.3	0.3	0.27	0.32
5	Mm.19904	NM_007527	Bcl2-associated X protein	Bax	0.25	0.18	0.19	0.18	0.25	0.21	0.3	0.29	0.27	0.3	0.34
6	Mm.257460	NM_009741	B-cell leukemia/lymphoma 2	Bcl2	0.69	0.98	0.78	0.97	0.8	0.86	0.83	0.56	0.77	0.85	1
7	Mm.378888	NM_007536	B-cell leukemia/lymphoma 2 related protein A1d	Bcl2a1d	0.25	0.38	0.23	0.22	0.21	0.35	0.46	0.2	0.22	0.89	0.53
8	Mm.238213	NM_009743	RIKEN cDNA A630035D09 gene	Bcl2l1	0.27	0.39	0.23	0.19	0.23	0.15	0.26	0.18	0.19	0.17	0.21
9	Mm.6898	NM_008670	Baculoviral IAP repeat-containing 1a	Birc1a	0.19	0.21	0.26	0.19	0.19	0.19	0.26	0.23	0.24	0.2	0.28
10	Mm.89961	NM_010872	Baculoviral IAP repeat-containing 1b	Birc1b	0.18	0.2	0.25	0.22	0.24	0.24	0.31	0.24	0.32	0.27	0.36
11	Mm.290476	NM_010870	Baculoviral IAP repeat-containing 1e	Birc1e	0.2	0.2	0.22	0.22	0.21	0.19	0.31	0.23	0.23	0.2	0.25
12	Mm.2026	NM_007464	Baculoviral IAP repeat-containing 3	Birc3	0.21	0.15	0.2	0.17	0.18	0.18	0.34	0.21	0.24	0.24	0.22
13	Mm.335659	NM_007465	Baculoviral IAP repeat-containing 2	Birc2	0.3	0.26	0.27	0.42	0.6	0.7	0.79	0.43	0.6	0.92	0.97
14	Mm.103205	NM_007553	Bone morphogenetic protein 2	Bmp2	0.28	0.37	0.23	0.23	0.23	0.2	0.32	0.21	0.22	0.19	0.24
15	Mm.6813	NM_007554	Bone morphogenetic protein 4	Bmp4	0.36	0.36	0.23	0.33	0.22	0.22	0.36	0.21	0.21	0.3	0.25
16	Mm.244975	NM_009764	Breast cancer 1	Brca1	0.44	0.5	0.37	0.27	0.39	0.33	0.44	0.28	0.33	0.26	0.33
17	Mm.273049	NM_007631	Cyclin D1	Ccnd1	0.73	0.98	0.76	1.13	1.11	0.96	1.13	0.79	1.02	0.99	1.04
18	Mm.111326	NM_016756	Cyclin-dependent kinase 2	Cdk2	0.23	0.21	0.3	0.21	0.25	0.3	0.35	0.33	0.31	0.35	0.43
19	Mm.195663	NM_007669	Cyclin-dependent kinase inhibitor 1A (P21)	Cdkn1a	0.54	0.99	0.33	0.47	0.62	0.74	0.91	0.61	0.99	0.79	0.91
20	Mm.2958	NM_009875	Cyclin-dependent kinase inhibitor 1B (P27)	Cdkn1b	0.35	0.31	0.36	0.5	0.39	0.33	0.47	0.3	0.4	0.29	0.46
21	Mm.168789	NM_009876	Cyclin-dependent kinase inhibitor 1C (P57)	Cdkn1c	0.27	0.22	0.22	0.23	0.25	0.27	0.39	0.27	0.29	0.26	0.35
22	Mm.4733	NM_009877	Cyclin-dependent kinase inhibitor 2A	Cdkn2a	0.97	1.04	0.72	0.54	0.72	0.91	1.05	0.37	0.91	0.92	1.02
23	Mm.269426	NM_007670	Cyclin-dependent kinase inhibitor 2B (p15, inhibits CDK4)	Cdkn2b	1.05	1.16	1.27	1.07	1.11	0.96	1.21	0.8	1	0.93	1.01
24	Mm.1912	NM_007671	Cyclin-dependent kinase inhibitor 2C (p18, inhibits CDK4)	Cdkn2c	1	0.98	0.51	0.89	0.95	0.97	1.18	0.65	0.91	0.93	1.02
25	Mm.29020	NM_009878	Cyclin-dependent kinase inhibitor 2D (p19, inhibits CDK4)	Cdkn2d	0.21	0.17	0.24	0.23	0.17	0.18	0.32	0.26	0.25	0.23	0.28
26	Mm.277735	NM_007742	Procollagen, type I, alpha 1	Col1a1	0.25	0.21	0.23	1.19	0.28	0.65	1.19	1.06	1.02	1	0.9
27	Mm.268737	NM_009972	Casein beta	Csn2	0.2	0.16	0.21	0.25	0.21	0.19	0.36	0.29	0.27	0.24	0.26
28	Mm.231395	NM_009983	Cathepsin D	Ctsd	0.75	0.32	0.41	0.61	0.55	0.88	1.01	0.56	0.71	1	0.95
29	Mm.766	NM_008599	Chemokine (C-X-C motif) ligand 9	Cxcl9	0.24	0.2	0.19	0.23	0.21	0.25	0.37	0.26	0.27	0.22	0.31
30	Mm.239041	NM_013642	Dual specificity phosphatase 1	Dusp1	0.76	0.51	0.33	0.84	0.29	0.35	1.21	0.9	0.99	0.85	0.45
31	Mm.14543	NM_010104	Endothelin 1	Edn1	0.29	0.41	0.27	0.25	0.23	0.21	0.44	0.24	0.26	0.2	0.22
32	Mm.8534	NM_007912	RIKEN cDNA E230015J15 gene	Egfr	0.27	0.35	0.23	0.26	0.21	0.25	0.83	0.29	0.42	0.21	0.27
33	Mm.181959	NM_007913	Early growth response 1	Egr1	0.88	0.32	0.47	1.25	0.57	0.25	1.14	0.79	0.45	0.56	0.41
34	Mm.250256	NM_015744	Ectonucleotide pyrophosphatase/phosphodiesterase 2	Enpp2	0.32	0.16	0.22	0.35	0.2	0.18	0.38	0.3	0.33	1	0.27
35	Mm.349116	NM_007942	Erythropoietin	Epo	0.28	0.19	0.24	0.28	0.26	0.21	0.43	0.33	0.32	0.27	0.29
36	Mm.193099	NM_010233	Fibronectin 1	Fn1	1.02	1.01	0.87	1.26	1.11	0.84	1.19	0.93	1.02	1	0.91
37	Mm.269137	NM_016770	Folate hydrolase	Folh1	0.28	0.24	0.2	0.24	0.23	0.21	0.35	0.3	0.27	0.24	0.23
38	Mm.246513	NM_010234	FBJ osteosarcoma related oncogene	Fos	0.36	0.48	0.3	0.25	0.29	0.2	0.44	0.27	0.28	0.2	0.26
39	Mm.6215	NM_010235	Fos-like antigen 1	Fosl1	0.72	0.63	0.32	0.3	0.33	0.58	0.6	0.35	0.53	0.38	0.76
40	Mm.938	NM_010446	Forkhead box A2	Foxa2	0.34	0.34	0.21	0.18	0.21	0.2	0.31	0.23	0.23	0.16	0.19
41	Mm.1236	NM_007836	Growth arrest and DNA-damage-inducible 45 alpha	Gadd45a	0.41	0.32	0.26	0.29	0.34	0.5	0.44	0.31	0.65	0.63	0.8
42	Mm.254493	NM_020259	Hedgehog-interacting protein	Hhip	0.35	0.34	0.34	0.26	0.29	0.25	0.41	0.39	0.35	0.29	0.36
43	Mm.3879	NM_010431	Hypoxia inducible factor 1, alpha subunit	Hif1a	0.74	0.49	0.52	0.56	0.9	0.46	1.13	0.84	1.01	0.89	1
44	Mm.196605	NM_010438	Hexokinase 1	Hk1	0.36	0.26	0.28	0.45	0.48	0.29	0.48	0.38	0.43	0.28	0.44
45	Mm.276389	NM_010442	Heme oxygenase (decycling) 1	Hmox1	0.67	0.33	0.25	0.32	0.27	0.4	0.91	0.65	0.88	0.39	0.43
46	Mm.347444	NM_008296	Heat shock factor 1	Hsf1	0.32	0.35	0.22	0.27	0.27	0.22	0.39	0.31	0.32	0.21	0.26
47	Mm.239865	NM_008300	Heat shock protein 4	Hspa4	0.92	1.13	0.6	0.51	1.01	0.47	1.16	0.85	0.93	0.41	0.53
48	Mm.330160	NM_022310	Heat shock 70kD protein 5 (glucose-regulated protein)	Hspa5	1.03	1.04	0.75	0.44	0.96	1.02	1.18	0.91	0.96	0.94	0.98
49	Mm.13849	NM_013560	Heat shock protein 1	Hspb1	0.28	0.22	0.26	0.24	0.25	0.39	0.39	0.32	0.45	0.4	0.69
50	Mm.1980	NM_024441	Heat shock protein 2	Hspb2	0.98	1.16	1.42	1.26	1.12	0.98	1.07	0.93	1.02	0.98	1.02
51	Mm.371565	NM_010480	Heat shock protein 1, alpha	Hsp90aa1	0.98	1.2	1.24	0.72	1.11	0.87	0.96	0.93	1.02	1	1.01
52	Mm.90364	NM_010493	H1 histone family, member 0	Icam1	0.24	0.23	0.25	0.23	0.28	0.22	0.32	0.3	0.29	0.21	0.28
53	Mm.34871	NM_010496	Inhibitor of DNA binding 2	Id2	1.06	1.19	1.19	1.05	1.12	1	1.21	0.9	1.02	0.98	1.05
54	Mm.29254	NM_008343	Insulin-like growth factor binding protein 3	Igfbp3	0.28	0.39	0.25	0.25	0.26	0.28	0.37	0.28	0.3	0.27	0.32
55	Mm.14190	NM_008366	Interleukin 2	Il2	0.42	0.39	0.26	0.2	0.24	0.19	0.28	0.23	0.25	0.17	0.21
56	Mm.276360	NM_021283	Interleukin 4	Il4	0.26	0.3	0.21	0.18	0.21	0.2	0.31	0.23	0.25	0.17	0.21
57	Mm.233802	NM_010557	Interleukin 4 receptor, alpha	Il4ra	0.17	0.16	0.21	0.16	0.17	0.14	0.41	0.21	0.45	0.2	0.22
58	Mm.105218	NM_008390	Interferon regulatory factor 1	Irf1	0.3	0.49	0.37	0.38	0.4	0.76	0.33	0.27	0.39	0.98	0.91
59	Mm.275071	NM_010591	Jun oncogene	Jun	0.27	0.22	0.26	0.2	0.24	0.21	0.28	0.26	0.27	0.2	0.27
60	Mm.245297	NM_008455	Kallikrein B, plasma 1	Klkb1	0.24	0.2	0.2	0.22	0.23	0.19	0.26	0.26	0.27	0.17	0.24
61	Mm.87787	NM_010735	Lymphotoxin A	Lta	0.25	0.37	0.28	0.24	0.24	0.23	0.27	0.25	0.27	0.18	0.26
62	Mm.1639	NM_008562	Myeloid cell leukemia sequence 1	Mcl1	0.81	0.55	0.32	0.32	0.51	0.36	0.71	0.45	0.57	0.53	0.52
63	Mm.22670	NM_010786	Transformed mouse 3T3 cell double minute 2	Mdm2	0.4	0.38	0.23	0.18	0.23	0.16	0.25	0.22	0.3	0.15	0.22

64	Mm.14126	NM_019471	Matrix metalloproteinase 10	Mmp10	0.23	0.32	0.18	0.15	0.18	0.21	0.24	0.19	0.25	0.57	0.21
65	Mm.4825	NM_010810	Matrix metalloproteinase 7	Mmp7	0.15	0.16	0.2	0.12	0.15	0.13	0.18	0.16	0.22	0.19	0.21
66	Mm.2444	NM_010849	Myelocytomatosis oncogene	Myc	0.23	0.15	0.21	0.22	0.19	0.16	0.23	0.22	0.25	0.17	0.29
67	Mm.73682	NM_022995	Transmembrane, prostate androgen induced RNA	Tmepai	0.46	0.39	0.32	0.28	0.36	0.59	0.41	0.36	0.64	0.46	0.74
68	Mm.256765	NM_008689	Ubiquitin-conjugating enzyme E2D 3 (UBC4/5 homolog, yeast)	Nfkb1	0.42	0.23	0.22	0.17	0.21	0.16	0.26	0.33	0.23	0.16	0.22
69	Mm.170515	NM_010907	Nuclear factor of kappa light chain gene enhancer in B-cells inhibitor, alpha	Nfkbia	0.35	0.39	0.23	0.18	0.21	0.18	0.27	0.24	0.22	0.18	0.22
70	Mm.16730	NM_010915	Nerve growth factor, alpha	Klk1b4	0.24	0.31	0.21	0.17	0.2	0.18	0.22	0.19	0.19	0.15	0.2
71	Mm.2893	NM_010927	Nitric oxide synthase 2, inducible, macrophage	Nos2	0.23	0.32	0.21	0.19	0.19	0.17	0.31	0.18	0.22	0.14	0.19
72	Mm.12798	NM_008829	Progesterone receptor	Pgr	0.2	0.27	0.18	0.14	0.18	0.15	0.47	0.17	0.23	0.12	0.17
73	Mm.3020	NM_011146	Peroxisome proliferator activated receptor gamma	Pparg	0.16	0.17	0.2	0.15	0.17	0.19	0.21	0.24	0.32	0.2	0.36
74	Mm.228798	NM_008957	Patched homolog 1	Ptch1	0.2	0.16	0.21	0.17	0.23	0.13	0.2	0.18	0.21	0.15	0.25
75	Mm.287037	NM_008958	Patched homolog 2	Ptch2	0.23	0.14	0.19	0.15	0.15	0.14	0.18	0.21	0.19	0.13	0.19
76	Mm.292547	NM_011198	Prostaglandin-endoperoxide synthase 2	Ptgs2	1.02	0.24	0.23	0.63	0.2	0.16	0.85	1.01	0.83	0.37	0.21
77	Mm.260144	NM_007376	Pregnancy zone protein	Pzp	0.35	0.76	0.35	0.19	0.62	0.23	0.3	0.3	0.31	0.25	0.32
78	Mm.21002	NM_011400	Solute carrier family 2 (facilitated glucose transporter), member 1	Slc2a1	0.42	0.44	0.31	0.21	0.26	0.26	0.38	0.25	0.31	0.22	0.32
79	Mm.332607	NM_009327	Transcription factor 1	Tcf1	0.31	0.55	0.28	0.18	0.48	0.65	0.33	0.24	0.49	0.33	0.99
80	Mm.1293	NM_013693	Tumor necrosis factor	Tnf	0.22	0.31	0.22	0.16	0.21	0.19	0.26	0.19	0.22	0.14	0.21
81	Mm.116683	NM_009397	Tumor necrosis factor, alpha-induced protein 3	Tnfaip3	0.18	0.18	0.21	0.11	0.14	0.13	0.19	0.17	0.21	0.17	0.22
82	Mm.193430	NM_020275	Tumor necrosis factor receptor superfamily, member 10b	Tnfrsf10b	0.21	0.15	0.19	0.12	0.15	0.1	0.16	0.2	0.2	0.13	0.17
83	Mm.1626	NM_007987	Fas (TNF receptor superfamily member)	Fas	0.2	0.15	0.19	0.15	0.14	0.12	0.18	0.19	0.22	0.17	0.17
84	Mm.1062	NM_009425	Tumor necrosis factor (ligand) superfamily, member 10	Tnfsf10	0.22	0.23	0.18	0.15	0.18	0.19	0.19	0.21	0.19	0.16	0.28
85	Mm.239514	NM_009421	Tnf receptor-associated factor 1	Traf1	0.81	0.81	0.41	0.61	0.7	0.95	0.9	0.55	0.93	0.76	0.99
86	Mm.37214	NM_133977	Transferrin	Trf	0.37	1.27	1.4	0.25	1.12	1.04	1.14	0.3	0.6	1	1.06
87	Mm.28683	NM_011638	Transferrin receptor	Tfrc	0.92	1.27	1.26	0.28	1.09	0.92	0.77	0.89	0.88	0.6	0.98
88	Mm.248445	NM_009546	Tripartite motif protein 25	Trim25	0.38	0.4	0.35	0.87	0.8	0.6	1.09	0.55	0.95	0.45	0.88
89	Mm.222	NM_011640	Transformation related protein 53	Trp53	0.29	0.19	0.24	0.5	0.33	0.25	0.44	0.53	0.51	0.49	0.52
90	Mm.76649	NM_011693	Vascular cell adhesion molecule 1	Vcam1	0.22	0.15	0.18	0.15	0.15	0.21	0.36	0.25	0.41	0.81	0.3
91	Mm.282184	NM_009505	Vascular endothelial growth factor A	Vegfa	0.42	0.18	0.2	0.17	0.16	0.18	0.31	0.28	0.3	0.22	0.22
92	Mm.35705	NM_009517	Wild-type p53-induced gene 1	Zmat3	0.33	0.28	0.21	0.2	0.22	0.25	0.26	0.26	0.35	0.22	0.3
93	Mm.10222	NM_018865	WNT1 inducible signaling pathway protein 1	Wisp1	0.28	0.3	0.21	0.19	0.22	0.25	0.25	0.22	0.26	0.22	0.3
94	Mm.1123	NM_021279	Wingless-related MMTV integration site 1	Wnt1	0.26	0.42	0.32	0.21	0.28	0.27	0.27	0.23	0.23	0.21	0.29
95	Mm.33653	NM_023653	Wingless-related MMTV integration site 2	Wnt2	0.39	0.41	0.35	0.27	0.3	0.3	0.35	0.32	0.28	0.23	0.32
96	Mm.307022	NM_019653	WD repeat and SOCS box-containing 1	Wsb1	0.81	0.51	0.42	0.36	0.46	0.43	0.81	0.53	0.71	0.58	0.52
