# Supplementary material for: Mesenchymal/Stromal Gene Expression Signature Relates to Basal-Like Breast Cancers, Identifies Bone Metastasis and Predicts Resistance to Therapies
Source: PLoS One. 2010 Nov 30;5(11):e14131. doi: 10.1371/journal.pone.0014131 (PMC2994727; doi:10.1371/journal.pone.0014131)
Supplement: Table S3 — Reference source of publicly available human microarray datasets. (0.08 MB PDF) [file pone.0014131.s003.pdf]

Description	Number of samples	Accession number	Database
**Prostate Cancer**	**19**	**E-GEOD-3325**	Arrayexpress project: http://www.ebi.ac.uk/microarray-as/ae/
**Bladder Cancer**	**12**	**E-GEOD-7476**	Arrayexpress project: http://www.ebi.ac.uk/microarray-as/ae/
**Breast Tumor vs. Normal Tissue**	**62**	**E-GEOD-7904**	Arrayexpress project: http://www.ebi.ac.uk/microarray-as/ae/
**Breast Tumors**	**129**	**E-GEOD-5460**	Arrayexpress project: http://www.ebi.ac.uk/microarray-as/ae/
**MSC**	**11**	**E-GEOD-9764**	Arrayexpress project: http://www.ebi.ac.uk/microarray-as/ae/
**Gastrointestinal Tumor**	**32**	**E-GEOD-8167**	Arrayexpress project: http://www.ebi.ac.uk/microarray-as/ae/
**Glioma**	**180**	**E-GEOD-4290**	Arrayexpress project: http://www.ebi.ac.uk/microarray-as/ae/
**Head and Neck Carcinoma**	**47**	**E-TABM-302**	Arrayexpress project: http://www.ebi.ac.uk/microarray-as/ae/
**Cancer Cell Lines**	**13**	**E-GEOD-10709**	Arrayexpress project: http://www.ebi.ac.uk/microarray-as/ae/
**Invasive edge, core thyroid carcinoma**	**18**	**E-GEOD-6004**	Arrayexpress project: http://www.ebi.ac.uk/microarray-as/ae/
**Breast Tumor, Breast Normal Tissue**	**30**	**E-GEOD-5764**	Arrayexpress project: http://www.ebi.ac.uk/microarray-as/ae/
**Lung Cancer**	**111**	**E-GEOD-3141**	Arrayexpress project: http://www.ebi.ac.uk/microarray-as/ae/
**Melanoma**	**87**	**E-GEOD-7553**	Arrayexpress project: http://www.ebi.ac.uk/microarray-as/ae/
**MSC**	**13**	**E-GEOD-9593**	Arrayexpress project: http://www.ebi.ac.uk/microarray-as/ae/
**MSC**	**30**	**E-GEOD-7637**	Arrayexpress project: http://www.ebi.ac.uk/microarray-as/ae/
**MSCs**	**18**	**E-MEXP-563**	Arrayexpress project: http://www.ebi.ac.uk/microarray-as/ae/
**Ovarian Cancer**	**27**	**E-MEXP-935**	Arrayexpress project: http://www.ebi.ac.uk/microarray-as/ae/
**pediatrix tumor**	**12**	**E-GEOD-8596**	Arrayexpress project: http://www.ebi.ac.uk/microarray-as/ae/
**Stromal samples from Carcinoma**	**22**	**E-GEOD-8977**	Arrayexpress project: http://www.ebi.ac.uk/microarray-as/ae/
**Tumor Collection from IGC and expO archive:**	**1973**	**E-GEOD-2109**	IGC and ExpO project: https://expo.intgen.org/geo/home.do
including	**breast**		
	**lung**		
	**brain**		
	**bladder**		
	**prostate**		
	**pancreas**		
	**liver**		
	**kidney**		
	**ovary**		
	**endometrium**		
	**others**		
