# Supplementary material for: Mesenchymal/Stromal Gene Expression Signature Relates to Basal-Like Breast Cancers, Identifies Bone Metastasis and Predicts Resistance to Therapies
Source: PLoS One. 2010 Nov 30;5(11):e14131. doi: 10.1371/journal.pone.0014131 (PMC2994727; doi:10.1371/journal.pone.0014131)
Supplement: Table S4 — Percentage fraction of samples overexpressing Ptgs2. (0.05 MB PDF) [file pone.0014131.s004.pdf]

A

***Ptgs2* values higher than 4-fold the *Ptgs2* median value across the samples:**

**Histotype**	**Total number of samples**	**Number of samples with ptgs2 value > 3xwhole-genome-median within each dataset**	**Percentage fraction**
Breast	508	10	1.97
Sarcomas	57	4	7.02
Stroma	34	31	91.18
MSC	73	57	78.08
Lung	233	69	29.61
Brain	135	11	8.15
Bladder	44	18	40.91
Lymphoma	21	2	9.52
prostate	80	35	43.75
pancreas	19	4	21.05
liver	43	2	4.65
gastrointestinal tract	41	4	9.76
Kidney	264	9	3.41
ovary	223	7	3.14
endometrium	69	1	1.45
melanoma	68	15	22.06
others	967	167	17.27

B

***Ptgs2* values higher than 3-fold the whole-genome median value within the dataset of each sample:**

**Histotype**	**Total number of samples**	**Number of samples with ptgs2 value > 3xwhole-genome-median within each dataset**	**Percentage fraction**
Breast	508	25	4.92
Sarcomas	57	5	8.77
Stroma	34	33	97.06
MSC	73	43	58.90
Lung	233	75	32.19
Brain	135	31	22.96
Bladder	44	25	56.82
**Lymphoma**	21	2	9.52
**prostate**	80	37	46.25
**pancreas**	19	2	10.53
**liver**	43	2	4.65
**gastrointestinal tract**	41	32	78.05
**Kidney**	264	9	3.41
**ovary**	223	15	6.73
**endometrium**	69	1	1.45
**melanoma**	68	20	29.41
**others**	967	170	17.58
