# Supplementary material for: Mesenchymal/Stromal Gene Expression Signature Relates to Basal-Like Breast Cancers, Identifies Bone Metastasis and Predicts Resistance to Therapies
Source: PLoS One. 2010 Nov 30;5(11):e14131. doi: 10.1371/journal.pone.0014131 (PMC2994727; doi:10.1371/journal.pone.0014131)
Supplement: Table S5 — A17 signatures. (0.26 MB PDF) [file pone.0014131.s005.pdf]

1. A17-Angiogenesis Signature

A17 OVEREXPRESSED GENES (≥ MEDIAN VALUE=0.355)					HUMAN ORTHOLOGS	
**GENE SYMBOL**	**UNIGENE**	**REFSEQ**	**GENE DESCRIPTION**	**A17 EXPRESSION VALUE**	**GENE SYMBOL**	**GENE ID**
Adamts8	Mm.100582	NM_013906	A disintegrin-like and metalloprotease (reprolysin type) with thrombospondin type 1 motif, 8	0.73	ADAMTS8	11095
Rnase4	Mm.202665	NM_007447	Ribonuclease, RNase A family 4	0.79	RNASE4	6038
Cdh5	Mm.21767	NM_009868	Cadherin 5	0.65	CDH5	1003
Chga	Mm.4137	NM_007693	Chromogranin A	0.43	CHGA	1113
Ctgf	Mm.1810	NM_010217	Connective tissue growth factor	1.03	CTGF	1490
Efnb2	Mm.209813	NM_010111	RIKEN cDNA E230020D15 gene	0.4	EFNB2	1948
Eng	Mm.225297	NM_007932	Endoglin	0.36	ENG	2022
Ephb4	Mm.34533	NM_010144	Eph receptor B4	0.49	EPHB4	2050
Fgf16	Mm.154768	NM_030614	Fibroblast growth factor 16	0.45	FGF16	8823
Fgf7	Mm.330557	NM_008008	Fibroblast growth factor 7	0.59	FGF7	2252
Fgfr1	Mm.265716	NM_010206	Fibroblast growth factor receptor 1	1.03	FGFR1	2260
Flt1	Mm.3464	NM_010228	FMS-like tyrosine kinase 1	0.82	FLT1	2321
Fn1	Mm.193099	NM_010233	Fibronectin 1	0.95	FN1	2335
Cxcl1	Mm.21013	NM_008176	Chemokine (C-X-C motif) ligand 1	0.36	CXCL1	2919
Hgf	Mm.267078	NM_010427	Hepatocyte growth factor	0.57	HGF	3082
Hif1a	Mm.3879	NM_010431	Hypoxia inducible factor 1, alpha subunit	0.9	HIF1A	3091
Igf1	Mm.268521	NM_010512	Insulin-like growth factor 1	0.58	IGF1	3479
Itga5	Mm.16234	NM_010577	Integrin alpha 5 (fibronectin receptor alpha)	0.55	ITGA5	3678
Mmp2	Mm.29564	NM_008610	Matrix metalloproteinase 2	0.56	MMP2	4313
Msr1	Mm.239291	NM_031195	Macrophage scavenger receptor 1	0.91	MSR1	4481
Nos3	Mm.258415	NM_008713	Nitric oxide synthase 3, endothelial cell	0.49	NOS3	4846
Pdgfra	Mm.221403	NM_011058	Platelet derived growth factor receptor, alpha polypeptide	0.52	PDGFRA	5156
Pdgfrb	Mm.4146	NM_008809	Platelet derived growth factor receptor, beta polypeptide	0.4	PDGFRB	5159
Pecam1	Mm.343951	NM_008816	Platelet/endothelial cell adhesion molecule	0.45	PECAM1	5175
Plau	Mm.4183	NM_008873	Plasminogen activator, urokinase	0.37	PLAU	5328
Ptgs1	Mm.275434	NM_008969	Prostaglandin-endoperoxide synthase 1	0.36	PTGS1	5742
Ptgs2	Mm.292547	NM_011198	Prostaglandin-endoperoxide synthase 2	0.68	PTGS2	5743
Rsn	Mm.241109	NM_019765	Restin (Reed-Steinberg cell-expressed intermediate filament-associated protein)	0.37	CLIP1	6249
Serpine1	Mm.250422	NM_008871	Serine (or cysteine) proteinase inhibitor, clade E, member 1	0.39	SERPINE1	5054
Serpinb2	Mm.271870	NM_011111	Serine (or cysteine) proteinase inhibitor, clade B, member 2	0.47	SERPINB2	5055
Serpinf1	Mm.2044	NM_011340	Serine (or cysteine) proteinase inhibitor, clade F, member 1	0.76	SERPINF1	5176
Sparc	Mm.291442	NM_009242	Secreted acidic cysteine rich glycoprotein	0.76	SPARC	6678
Spp1	Mm.288474	NM_009263	Secreted phosphoprotein 1	0.84	SPP1	6696
Tek	Mm.14313	NM_013690	Endothelial-specific receptor tyrosine kinase	0.72	TEK	7010
Tgfa	Mm.137222	NM_031199	Transforming growth factor alpha	0.5	TGFA	7039
Tgfb3	Mm.307887	NM_009368	Transforming growth factor, beta 3	0.82	TGFB3	7043
Tgfbr1	Mm.197552	NM_009370	Transforming growth factor, beta receptor I	0.63	TGFBR1	7046
Tgfbr2	Mm.172346	NM_009371	Transforming growth factor, beta receptor II	0.44	TGFBR2	7048

Tgfbr3	Mm.200775	NM_011578	Similar to Ornithine decarboxylase (ODC)	0.38	TGFBR3	7049
Thbs1	Mm.4159	NM_011580	Thrombospondin 1	0.89	THBS1	7057
Thbs2	Mm.26688	NM_011581	Thrombospondin 2	0.75	THBS2	7058
Thbs3	Mm.2114	NM_013691	Thrombospondin 3	0.48	THBS3	7059
Timp1	Mm.8245	NM_011593	Tissue inhibitor of metalloproteinase 1	0.77	TIMP1	7076
Timp2	Mm.206505	NM_011594	Tissue inhibitor of metalloproteinase 2	0.82	TIMP2	7077
Vcam1	Mm.76649	NM_011693	Vascular cell adhesion molecule 1	0.84	VCAM1	7412
Vegfa	Mm.282184	NM_009505	Vascular endothelial growth factor A	0.37	VEGFA	7422
Vegfb	Mm.15607	NM_011697	Vascular endothelial growth factor B	0.38	VEGFB	7423
Vegfc	Mm.1402	NM_009506	Vascular endothelial growth factor C	0.52	VEGFC	7424

2. A17-Stemness Signature

A17 OVEREXPRESSED GENES (≥ MEDIAN VALUE=0.27)					HUMAN ORTHOLOGS	
**GENE SYMBOL**	**UNIGENE**	**REFSEQ**	**GENE DESCRIPTION**	**A17 EXPRESSION VALUE**	**GENE SYMBOL**	**GENE ID**
Abcg2	Mm.333096	NM_011920	ATP-binding cassette, sub-family G (WHITE), member 2	0.31	ABCG2	9429
Acta2	Mm.213025	NM_007392	Actin, alpha 2, smooth muscle, aorta	0.99	ACTA2	59
Actc1	Mm.686	NM_009608	Actin, alpha, cardiac	0.51	ACTC1	70
Actg2	Mm.292865	NM_009610	Actin, gamma 2, smooth muscle, enteric	0.99	ACTG2	72
Acvr1	Mm.689	NM_007394	Activin A receptor, type 1	0.29	ACVR1	90
Acvr2	Mm.314338	NM_007396	Activin receptor IIA	0.49	ACVR2A	92
Nppa	Mm.19961	NM_008725	Natriuretic peptide precursor type A	0.38	NPPA	4878
Bdnf	Mm.1442	NM_007540	Brain derived neurotrophic factor	0.30	BDNF	627
Bmp1	Mm.27757	NM_009755	Bone morphogenetic protein 1	0.45	BMP1	649
Bmp10	Mm.57171	NM_009756	Bone morphogenetic protein 10	0.30	BMP10	27302
Bmp2	Mm.103205	NM_007553	Bone morphogenetic protein 2	0.32	BMP2	650
Bmp3	Mm.209571	NM_173404	Bone morphogenetic protein 3	1.09	BMP3	651
Bmp4	Mm.6813	NM_007554	Bone morphogenetic protein 4	0.41	BMP4	652
Bmp6	Mm.374781	NM_007556	Bone morphogenetic protein 6	0.79	BMP6	654
Bmp8a	Mm.318417	NM_007558	Bone morphogenetic protein 8a	0.38	BMP8A	353500
Bmpr1a	Mm.237825	NM_009758	Bone morphogenetic protein receptor, type 1A	0.70	BMPR1A	657
Bmpr1b	Mm.39089	NM_007560	Bone morphogenetic protein receptor, type 1B	0.33	BMPR1B	658
Bmpr2	Mm.7106	NM_007561	Bone morphogenic protein receptor, type II (serine/threonine kinase)	0.70	BMPR2	659
Ctnna1	Mm.18962	NM_009818	Catenin (cadherin associated protein), alpha 1	0.83	CTNNA1	1495
Catna2	Mm.34637	NM_009819	Catenin (cadherin associated protein), alpha 2	0.45	CTNNA2	1496
Catnal1	Mm.218891	NM_018761	Catenin (cadherin associated protein), alpha-like 1	0.59	CTNNAL1	8727
Ctnnb1	Mm.291928	NM_007614	Catenin (cadherin associated protein), beta 1	0.79	CTNNB1	1499
Catnd2	Mm.321648	NM_008729	Catenin (cadherin associated protein), delta 2	0.51	CTNND2	1501
Ccng2	Mm.3527	NM_007635	Cyclin G2	0.30	CCNG2	901
Cd34	Mm.29798	NM_133654	CD34 antigen	0.34	CD34	947
Cd44	Mm.330428	M27130	CD44 antigen	0.61	CD44	960
Cdh15	Mm.1976	NM_007662	Cadherin 15	0.38	CDH15	1013
Cdh2	Mm.257437	NM_007664	Cadherin 2	0.34	CDH2	1000

Cdh3	Mm.4658	NM_001037809	Cadherin 3	0.35	CDH3	1001
Cdh5	Mm.21767	NM_009868	Cadherin 5	0.89	CDH5	1003
Cdkn1a	Mm.195663	NM_007669	Cyclin-dependent kinase inhibitor 1A (P21)	0.78	CDKN1A	1026
Cdkn1b	Mm.2958	NM_009875	Cyclin-dependent kinase inhibitor 1B (P27)	0.29	CDKN1B	1027
Cer1	Mm.6780	NM_009887	Cerberus 1 homolog (Xenopus laevis)	0.32	CER1	9350
Cnp1	Mm.15711	NM_009923	Cyclic nucleotide phosphodiesterase 1	1.07	CNP	1267
Zfp91	Mm.290924	XM_909506	Zinc finger protein 91	1.04	ZFP91	80829
Cntfr	Mm.272210	NM_016673	Ciliary neurotrophic factor receptor	0.33	CNTFR	1271
Col6a2	Mm.1949	NM_146007	Procollagen, type VI, alpha 2	0.93	COL6A2	12834
Cst3	Mm.4263	NM_009976	Cystatin C	0.53	CST3	1471
Dnmt1	Mm.128580	NM_010066	DNA methyltransferase (cytosine-5) 1	0.76	DNMT1	1786
Egfr	Mm.8534	NM_007912	Epidermal growth factor receptor	0.67	EGFR	1956
Erbb2ip	Mm.277354	NM_021563	Erbb2 interacting protein	0.73	ERBB2IP	55914
Erbb3	Mm.29023	NM_010153	V-erb-b2 erythroblastic leukemia viral oncogene homolog 3 (avian)	0.75	ERBB3	2065
Erbb4	Mm.336982	XM_136682	V-erb-a erythroblastic leukemia viral oncogene homolog 4 (avian)	0.42	ERBB4	2066
Fabp4	Mm.582	NM_024406	Fatty acid binding protein 4, adipocyte	0.28	FABP4	2167
Fgf20	Mm.376109	NM_030610	Fibroblast growth factor 20	0.34	FGF20	26281
Fgf22	Mm.154211	NM_023304	Fibroblast growth factor 22	0.69	FGF22	27006
Fgf23	Mm.347933	NM_022657	Fibroblast growth factor 23	0.71	FGF23	8074
Fgf3	Mm.4947	NM_008007	Fibroblast growth factor 3	1.05	FGF3	2248
Fgf4	Mm.4956	NM_010202	Fibroblast growth factor 4	0.27	FGF4	2249
Fgf5	Mm.5055	NM_010203	Fibroblast growth factor 5	0.97	FGF5	2250
Fgfr1	Mm.265716	NM_010206	Fibroblast growth factor receptor 1	0.83	FGFR1	2260
Foxm1	Mm.42148	NM_008021	Forkhead box M1	0.45	FOXM1	2305
Foxo1	Mm.29891	NM_019739	Forkhead box O1	0.44	FOXO1	2308
Fzd1	Mm.246003	NM_021457	Frizzled homolog 1 (Drosophila)	0.84	FZD1	8321
Fzd7	Mm.297906	NM_008057	Frizzled homolog 7 (Drosophila)	0.35	FZD7	8324
Fzd8	Mm.184289	NM_008058	Frizzled homolog 8 (Drosophila)	0.76	FZD8	8325
Gdf1	Mm.258280	NM_008107	Growth differentiation factor 1	0.46	GDF1	2657
Gdf5	Mm.4744	NM_008109	Growth differentiation factor 5	0.30	GDF5	8200
Gdf8	Mm.3514	NM_010834	Growth differentiation factor 8	0.28	GDF8	2660
Gdf9	Mm.9714	NM_008110	Growth differentiation factor 9	0.56	GDF9	2661
Gjb1	Mm.21198	NM_008124	Gap junction membrane channel protein beta 1	0.58	GJB1	2705
Gjb5	Mm.26859	NM_010291	Gap junction membrane channel protein beta 5	0.53	GJB5	2709
Icam5	Mm.4629	NM_008319	Intercellular adhesion molecule 5, telencephalin	0.60	ICAM5	7087
Igf1r	Mm.275742	NM_010513	Insulin-like growth factor I receptor	0.64	IGF1R	3480
Igf2r	Mm.26553	NM_010515	Insulin-like growth factor 2 receptor	0.28	IGF2R	3482
Igfbp3	Mm.29254	NM_008343	Insulin-like growth factor binding protein 3	0.32	IGFBP3	3486
Il6	Mm.1019	NM_031168	Interleukin 6	0.92	IL6	3569
Il6ra	Mm.2856	NM_010559	Interleukin 6 receptor, alpha	0.30	IL6R	3570
Il6st	Mm.4364	NM_010560	Interleukin 6 signal transducer	0.91	IL6ST	3572
Ina	Mm.276251	NM_146100	Internexin neuronal intermediate filament protein, alpha	0.27	INA	9118

Inhba	Mm.8042	NM_008380	Inhibin beta-A	0.65	INHBA	3624
Inhbb	Mm.3092	NM_008381	Inhibin beta-B	0.78	INHBB	3625
Isl1	Mm.42242	NM_021459	ISL1 transcription factor, LIM/homeodomain (islet 1)	0.50	ISL1	3670
Itga5	Mm.16234	NM_010577	Integrin alpha 5 (fibronectin receptor alpha)	0.51	ITGA5	3678
Itga6	Mm.225096	NM_008397	Integrin alpha 6	0.34	ITGA6	3655
Itga7	Mm.179747	NM_008398	Integrin alpha 7	0.66	ITGA7	3679
Itgae	Mm.96	NM_008399	Integrin, alpha E, epithelial-associated	0.42	ITGAE	3682
Itgam	Mm.262106	NM_008401	Integrin alpha M	0.28	ITGAM	3684
Itgav	Mm.227	NM_008402	Integrin alpha V	0.77	ITGAV	3685
Itgax	Mm.22378	NM_021334	Integrin alpha X	0.43	ITGAX	3687
Itgb1	Mm.263396	NM_010578	Integrin beta 1 (fibronectin receptor beta)	0.79	ITGB1	3688
Itgb4	Mm.213873	L04678	Integrin beta 4	0.59	ITGB4	3691
Itgb5	Mm.6424	NM_010580	Integrin beta 5	1.03	ITGB5	3693
Itgb7	Mm.58	NM_013566	Integrin beta 7	0.27	ITGB7	3695
F11r	Mm.294882	NM_172647	F11 receptor	0.37	F11R	50848
Krt1-14	Mm.6974	NM_016958	Keratin complex 1, acidic, gene 14	0.31	KRT14	3861
Krt1-15	Mm.38498	NM_008469	Keratin complex 1, acidic, gene 15	0.55	KRT15	3866
Krt1-5	Mm.306829	XM_894886	Keratin complex 1, acidic, gene 5	0.46	KRT5	3852
Krt2-8	Mm.289759	NM_031170	Keratin complex 2, basic, gene 8	0.45	KRT8	3856
Lif	Mm.4964	NM_008501	Leukemia inhibitory factor	0.29	LIF	3976
Myh6	Mm.290003	NM_010856	Myosin, heavy polypeptide 6, cardiac muscle, alpha	0.60	MYH6	4624
Myl4	Mm.247636	NM_010858	Myosin, light polypeptide 4	0.66	MYL4	4635
Ncam1	Mm.4974	NM_010875	Neural cell adhesion molecule 1	0.51	NCAM1	4684
Nes	Mm.331129	NM_016701	Nestin	0.49	NES	10763
Nefl	Mm.1956	NM_010910	Neurofilament, light polypeptide	0.32	NEFL	4747
Ngfb	Mm.1259	NM_013609	Nerve growth factor, beta	1.06	NGFB	4803
Ngfr	Mm.283893	NM_033217	Nerve growth factor receptor (TNFR superfamily, member 16)	0.31	NGFR	4804
Nkx2-2	Mm.330639	NM_010919	NK2 transcription factor related, locus 2 (Drosophila)	0.99	NKX2-2	4821
Nkx2-5	Mm.41974	NM_008700	NK2 transcription factor related, locus 5 (Drosophila)	0.30	NKX2-5	1482
Nodal	Mm.57195	NM_013611	Nodal	0.41	NODAL	4838
Notch3	Mm.4945	NM_008716	Notch gene homolog 3 (Drosophila)	0.34	NOTCH3	4854
Notch4	Mm.173813	NM_010929	Notch gene homolog 4 (Drosophila)	0.85	NOTCH4	4855
A830055N07Rik	Mm.255566	NM_008734	RIKEN cDNA A830055N07 gene	0.28	NRG3	10718
Ntrk3	Mm.33496	NM_008746	Neurotrophic tyrosine kinase, receptor, type 3	0.42	NTRK3	4916
Numb	Mm.4390	NM_010949	Numb gene homolog (Drosophila)	0.31	NUMB	8650
Olig1	Mm.39300	NM_016968	Oligodendrocyte transcription factor 1	0.88	OLIG1	116448
Pdgfb	Mm.144089	NM_011057	Platelet derived growth factor, B polypeptide	0.35	PDGFB	5155
Pou3f2	Mm.129387	NM_008899	POU domain, class 3, transcription factor 2	1.07	POU3F2	5454
Pou3f3	Mm.40572	NM_008900	POU domain, class 3, transcription factor 3	0.96	POU3F3	5455
Pou5f1	Mm.17031	NM_013633	POU domain, class 5, transcription factor 1	0.32	POU5F1	5460
Pou6f1	Mm.28825	NM_010127	POU domain, class 6, transcription factor 1	0.27	POU6F1	5463
Grem2	Mm.25760	NM_011825	Gremlin 2 homolog, cysteine knot superfamily (Xenopus laevis)	0.34	GREM2	64388

Ptch1	Mm.228798	NM_008957	Patched homolog 1	0.70	PTCH1	5727
Pten	Mm.245395	NM_008960	Phosphatase and tensin homolog	0.78	PTEN	5728
S100b	Mm.235998	NM_009115	S100 protein, beta polypeptide, neural	0.70	S100B	6285
Shh	Mm.57202	NM_009170	Sonic hedgehog	0.61	SHH	6469
Slc2a1	Mm.21002	NM_011400	Solute carrier family 2 (facilitated glucose transporter), member 1	0.68	SLC2A1	6513
Snai1	Mm.2093	NM_011427	Snail homolog 1 (Drosophila)	0.33	SNAI1	6615
Snai2	Mm.4272	NM_011415	Snail homolog 2 (Drosophila)	0.57	SNAI2	6591
Sox1	Mm.39088	NM_009233	SRY-box containing gene 1	0.82	SOX1	6656
Sox3	Mm.35784	NM_009237	SRY-box containing gene 3	0.68	SOX3	6658
Sox4	Mm.240627	NM_009238	SRY-box containing gene 4	0.57	SOX4	6659
Tebp	Mm.305816	NM_019766	Prostaglandin E synthase 3 (cytosolic)	0.81	TEBP	7080
Tep1	Mm.318736	NM_009351	Telomerase associated protein 1	0.42	TEP1	7011
Terf1	Mm.4306	NM_009352	Telomeric repeat binding factor 1	0.30	TERF1	7013
Tgfb3	Mm.3992	NM_009368	Transforming growth factor, beta 3	0.39	TGFB3	7043
Tgfbr1	Mm.197552	NM_009370	Transforming growth factor, beta receptor I	0.47	TGFBR1	7046
Tgfbr2	Mm.172346	NM_009371	Transforming growth factor, beta receptor II	0.37	TGFBR2	7048
Vcam1	Mm.76649	NM_011693	Vascular cell adhesion molecule 1	0.43	VCAM1	7412
Vegfa	Mm.282184	NM_009505	Vascular endothelial growth factor A	0.43	VEGFA	7422
Vim	Mm.268000	NM_011701	Vimentin	0.34	VIM	7431
Zfp110	Mm.292297	NM_022981	Zinc finger protein 110	0.56	ZFP91-CNTF	386607

3. A17-Signal Transduction Signature

A17 OVEREXPRESSED GENES (≥ MEDIAN VALUE=0.27)					HUMAN ORTHOLOGS	
**GENE SYMBOL**	**UNIGENE**	**REFSEQ**	**GENE DESCRIPTION**	**A17 EXPRESSION VALUE**	**GENE SYMBOL**	**GENE ID**
Atf2	Mm.209903	NM_009715	Activating transcription factor 2	0.28	ATF2	1386
Bcl2	Mm.257460	NM_009741	B-cell leukemia/lymphoma 2	0.83	BCL2	596
Birc2	Mm.335659	NM_007465	Baculoviral IAP repeat-containing 2	0.36	BIRC2	329
Bmp4	Mm.6813	NM_007554	Bone morphogenetic protein 4	0.345	BMP4	652
Brca1	Mm.244975	NM_009764	Breast cancer 1	0.355	BRCA1	672
Ccnd1	Mm.273049	NM_007631	Cyclin D1	0.93	CCND1	595
Cdkn1a	Mm.195663	NM_007669	Cyclin-dependent kinase inhibitor 1A (P21)	0.505	CDKN1A	1026
Cdkn1b	Mm.2958	NM_009875	Cyclin-dependent kinase inhibitor 1B (P27)	0.425	CDKN1B	1027
Cdkn2a	Mm.4733	NM_009877	Cyclin-dependent kinase inhibitor 2A	0.755	CDKN2A	1029
Cdkn2b	Mm.269426	NM_007670	Cyclin-dependent kinase inhibitor 2B (p15, inhibits CDK4)	1.06	CDKN2B	1030
Cdkn2c	Mm.1912	NM_007671	Cyclin-dependent kinase inhibitor 2C (p18, inhibits CDK4)	0.945	CDKN2C	1031
Col1a1	Mm.277735	NM_007742	Procollagen, type I, alpha 1	0.72	COL1A1	1277
Ctsd	Mm.231395	NM_009983	Cathepsin D	0.68	CTSD	1509
Dusp1	Mm.239041	NM_013642	Dual specificity phosphatase 1	0.8	DUSP1	1843
Edn1	Mm.14543	NM_010104	Endothelin 1	0.27	EDN1	1906
Egr1	Mm.181959	NM_007913	Early growth response 1	1.065	EGR1	1958
Enpp2	Mm.250256	NM_015744	Ectonucleotide pyrophosphatase/phosphodiesterase 2	0.335	ENPP2	5168
Epo	Mm.349116	NM_007942	Erythropoietin	0.28	EPO	2056

Fn1	Mm.193099	NM_010233	Fibronectin 1	1.14	FN1	2335
Fos	Mm.246513	NM_010234	FBJ osteosarcoma related oncogene	0.305	FOS	2353
Fosl1	Mm.6215	NM_010235	Fos-like antigen 1	0.51	FOSL1	8061
Gadd45a	Mm.1236	NM_007836	Growth arrest and DNA-damage-inducible 45 alpha	0.35	GADD45A	1647
Hhip	Mm.254493	NM_020259	Hedgehog-interacting protein	0.305	HHIP	64399
Hif1a	Mm.3879	NM_010431	Hypoxia inducible factor 1, alpha subunit	0.65	HIF1A	3091
Hk1	Mm.196605	NM_010438	Hexokinase 1	0.405	HK1	3098
Hmox1	Mm.276389	NM_010442	Heme oxygenase (decycling) 1	0.495	HMOX1	3162
Hsf1	Mm.347444	NM_008296	Heat shock factor 1	0.295	HSF1	3297
Hspa4	Mm.239865	NM_008300	Heat shock protein 4	0.715	HSPA4	3308
Hspa5	Mm.330160	NM_022310	Heat shock 70kD protein 5 (glucose-regulated protein)	0.735	HSPA5	3309
Hspb2	Mm.1980	NM_024441	Heat shock protein 2	1.12	HSPB2	3316
Hspca	Mm.371565	NM_010480	Heat shock protein 1, alpha	0.85	HSPCA	3320
Idb2	Mm.34871	NM_010496	Inhibitor of DNA binding 2	1.055	IDB2	15902
Il2	Mm.14190	NM_008366	Interleukin 2	0.31	IL2	3558
Irf1	Mm.105218	NM_008390	Interferon regulatory factor 1	0.34	IRF1	3659
Mcl1	Mm.1639	NM_008562	Myeloid cell leukemia sequence 1	0.565	MCL1	4170
Mdm2	Mm.22670	NM_010786	Transformed mouse 3T3 cell double minute 2	0.29	MDM2	4193
Tmepai	Mm.73682	NM_022995	Transmembrane, prostate androgen induced RNA	0.37	PMEPA1	56937
Nfkb1	Mm.256765	NM_008689	Ubiquitin-conjugating enzyme E2D 3 (UBC4/5 homolog, yeast)	0.295	NFKB1	4790
Ptgs2	Mm.292547	NM_011198	Prostaglandin-endoperoxide synthase 2	0.825	PTGS2	5743
Pzp	Mm.260144	NM_007376	Pregnancy zone protein	0.27	PZP	5858
Slc2a1	Mm.21002	NM_011400	Solute carrier family 2 (facilitated glucose transporter), member 1	0.315	SLC2A1	6513
Traf1	Mm.239514	NM_009421	Tnf receptor-associated factor 1	0.71	TRAF1	7185
Trf	Mm.37214	NM_133977	Transferrin	0.31	TERF1	7013
Tfrc	Mm.28683	NM_011638	Transferrin receptor	0.6	TFRC	7037
Trim25	Mm.248445	NM_009546	Tripartite motif protein 25	0.625	TRIM25	7706
Trp53	Mm.222	NM_011640	Transformation related protein 53	0.395	TRP53	7157
Vegfa	Mm.282184	NM_009505	Vascular endothelial growth factor A	0.295	VEGFA	7422
Wnt2	Mm.33653	NM_023653	Wingless-related MMTV integration site 2	0.33	WNT2	7472
Wsb1	Mm.307022	NM_019653	WD repeat and SOCS box-containing 1	0.585	WSB1	26118
