# Supplementary material for: Mesenchymal/Stromal Gene Expression Signature Relates to Basal-Like Breast Cancers, Identifies Bone Metastasis and Predicts Resistance to Therapies
Source: PLoS One. 2010 Nov 30;5(11):e14131. doi: 10.1371/journal.pone.0014131 (PMC2994727; doi:10.1371/journal.pone.0014131)
Supplement: Table S6 — Reference source of microarray dataset of samples undergone hormonal therapy. (0.05 MB PDF) [file pone.0014131.s006.pdf]

Accession Number	Number of samples	Description	Reference
E-GEOD -8139	51	Transcription profiling of human in vivo xenograft models of estrogen receptor-positive (ER+) breast cancer, with or without HER2 over-expression (MCF7/HER2-18 and MCF7 wt, respectively)	No reference
E-GEOD-8140 : va accoppiato allo 8139: contiene le cellule di controllo	14	Transcription profiling of mouse MCF7 wt xenografts	No reference
E-GEOD-8742	10	Transcription profiling of human cell lines MCF7, COLO-205 and SK-MEL-5 following treatment with PEP008	Sarah-Jane Cozzi, Peter G Parsons, Steven M Ogbourne, Julie Pedley, Glen M Boyle. *Cancer Res* 66(20):10083-91 (2006), PubMed 17047072
E-GEOD-7327	7	Transcription profiling of human tamoxifen-resistant MCF-7 wild-type murine xenograft tumors with E2-supplemented tumors MCF7 xenografts	Suleiman Massarweh, C Kent Osborne, Chad J Creighton, Lanfang Qin, Anna Tsimelzon, Shixia Huang, Heidi Weiss, Mothaffar Rimawi, Rachel Schiff. *Cancer Res* 68(3):826-33 (2008), PubMed 18245484
E-GEOD-10879	11	Transcription profiling of human hormone-responsive MCF-7 cells versus estrogen-deprived MCF-7:5C and MCF-7:2A breast cancer cells	No reference
