# Supplementary figures and images for: Mesenchymal/Stromal Gene Expression Signature Relates to Basal-Like Breast Cancers, Identifies Bone Metastasis and Predicts Resistance to Therapies
Source: PLoS One. 2010 Nov 30;5(11):e14131. doi: 10.1371/journal.pone.0014131 (PMC2994727; doi:10.1371/journal.pone.0014131)

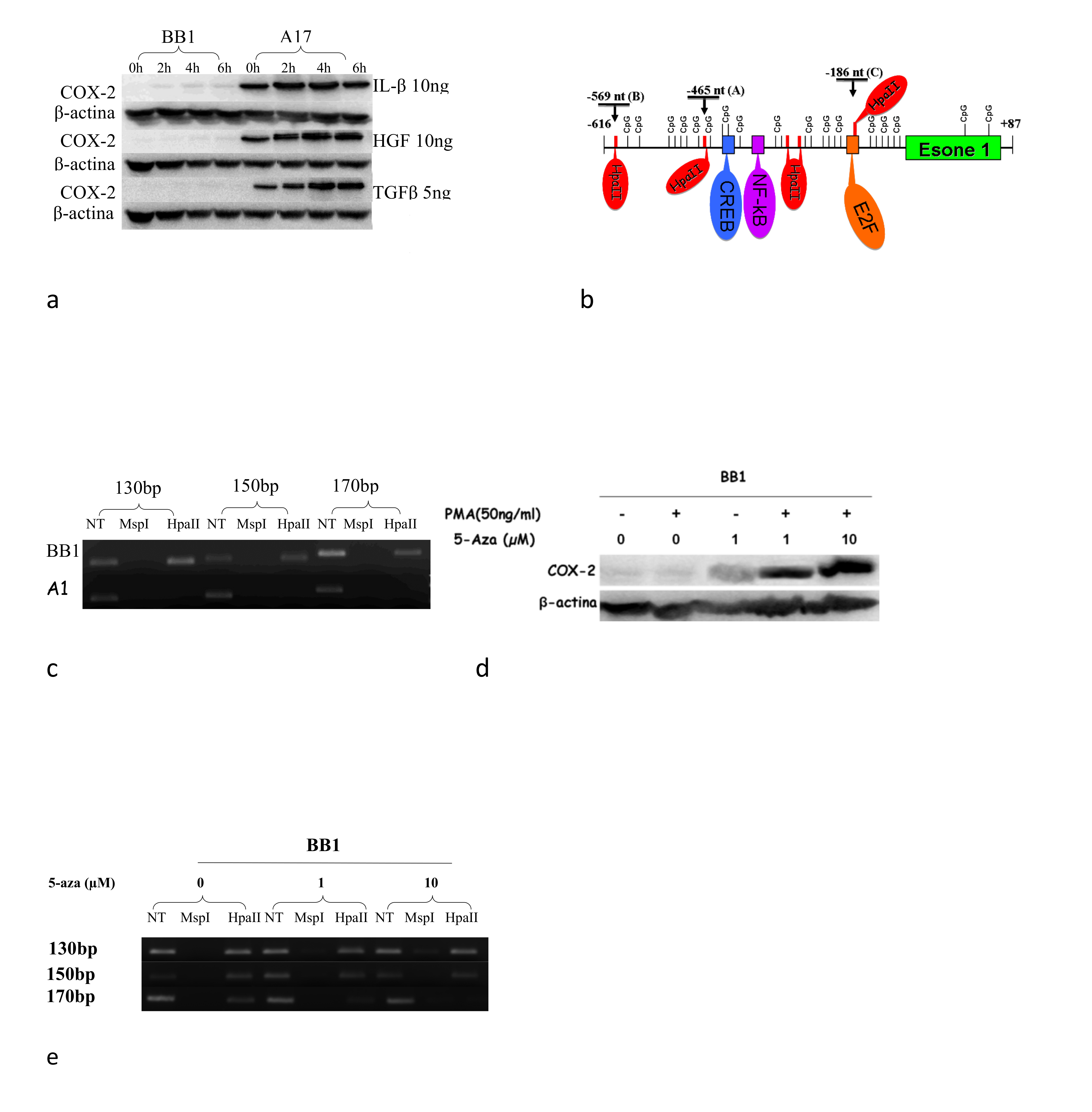

Supplement: Figure S1 — DNA Methylation pattern in the COX-2/PTGS2 promoter were determined by Methylation-Specific PCR (MSP). Treatment with IL-1β, HGF and TGFβ for 2, 4 and 6 hours, was not able to induce the COX-2 expression in BB1, while resulted in increased levels of COX2 in the A17 cell (a). Three primers were designed for amplifying the sequence containing three side CpG in the promoter of COX2: −465 nt (A), −569 nt (B) and −186 nt (C) (b and c). COX2 promoter resulted hypermethylated in BB1 cells, but it was demethylated in A17 cells (d). The state of demethylation of the promoter COX2 was examined by MSP after treatment of cells BB1 for 72 hours with the substance demethylating 5-aza-CdR at a concentration of 1 and 10 µM (e). (1.70 MB TIF) [file pone.0014131.s007.tif]

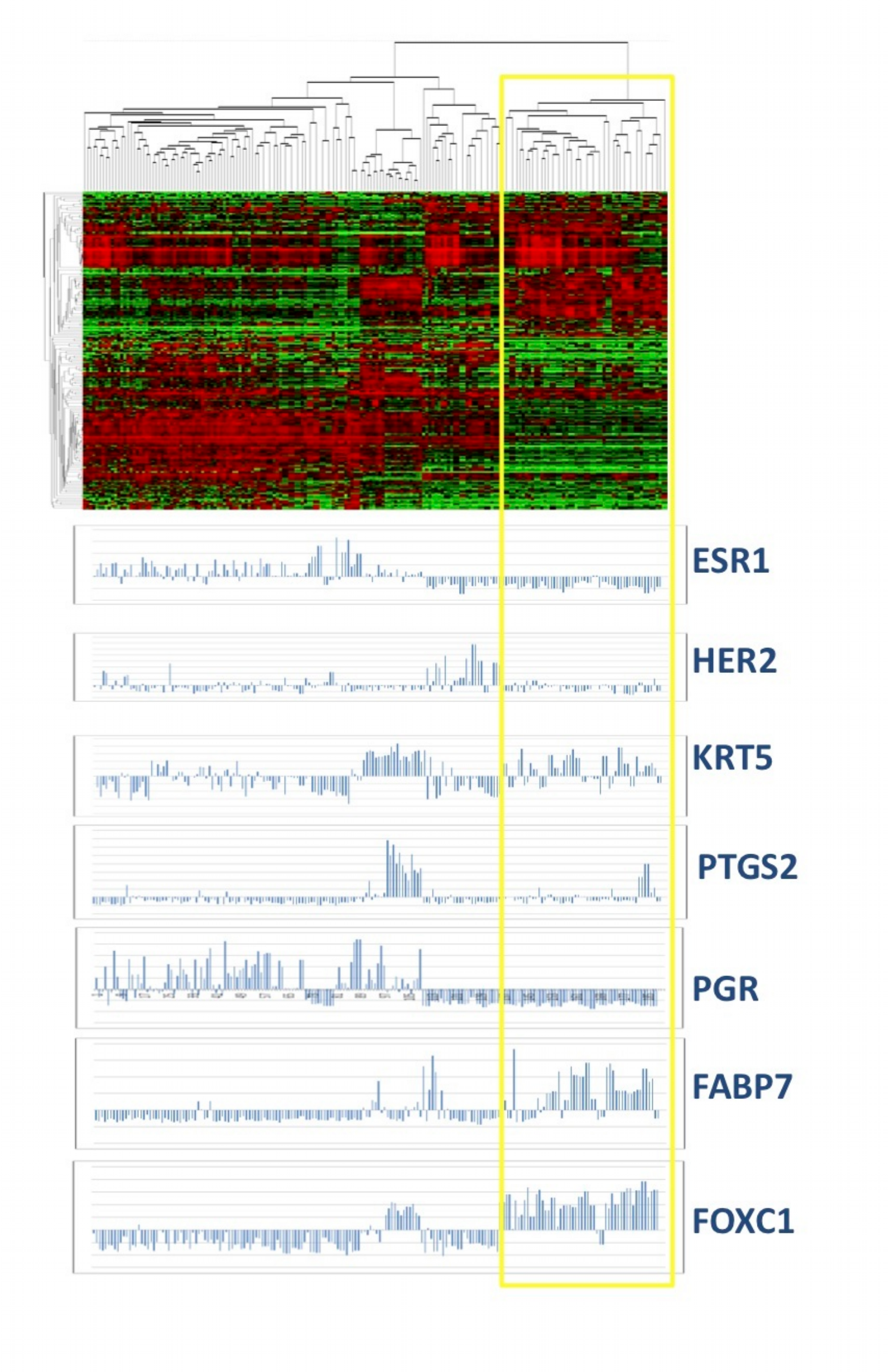

Supplement: Figure S2 — Preliminary unsupervised cluster analysis of the most differentially expressed genes (SD>1.8), restricted to breast cancers only, confirmed that the dataset previously described in figure 3 comprises all five subtypes, including the basal-like one (ER-negative, HER-2-negative, KRT5-positive) (yellow square). (8.20 MB TIF) [file pone.0014131.s008.tif]

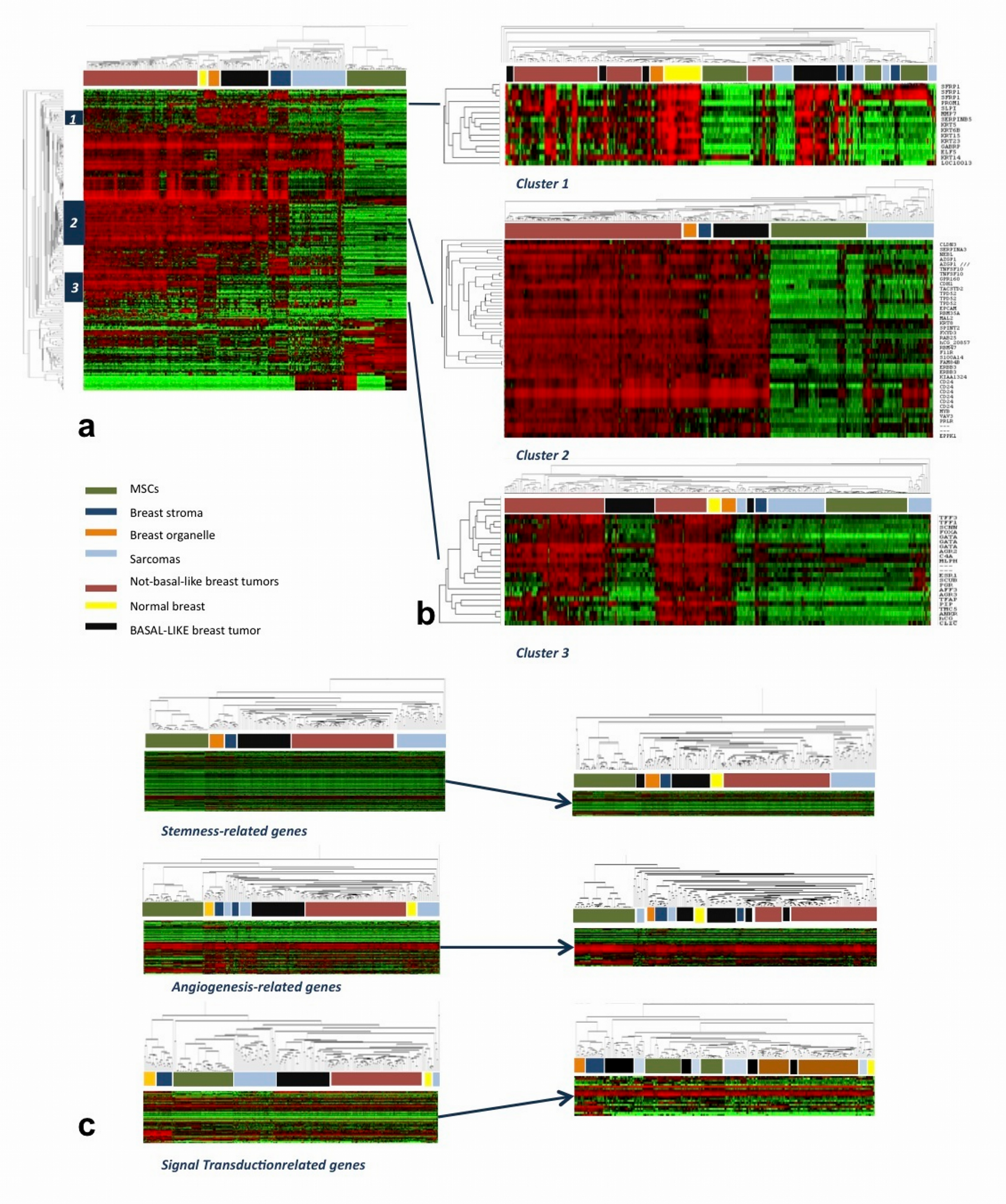

Supplement: Figure S3 — When breast tumors were clusterized together with mesenchymal samples (breast stroma, MSCs, sarcomas), basal-like samples clusterized with breast cancers, whereas breast stroma, MSCs and sarcomas formed distinct clusters (a). Thus, we supervised whole-genome clusters searching for any gene cluster that might relate basal-like to epithelial or mesenchymal samples. We identified three gene sets with an apparently interesting pattern of expression, and performed hierarchical clusterization of these genes separately (b). Cluster 1 comprised apparently peculiar genes of basal-like cells and normal breast/organelle samples. As expected, it included KRT5, KRT14 and KRT15. Hierarchical clusterization of these genes did not clearly separate epithelial and mesenchymal phenotypes, thus indicating that these genes failed to directly relate basal-like cells to non-basal breast cancer or mesenchymal phenotypes. Cluster 2 relates basal-like samples to non-basal breast cancers and breast stroma, whereas MSCs and Sarcomas formed distinct clusters. In Cluster 3, most of the basal-like samples fell into a large cluster which included all mesenchymal samples, along with a limited number of non-basal breast cancers. Finally we clusterized breast cancers and mesenchymal samples for the genes comprised in the stemness-, angiogenesis- and signal transduction-related arrays described above, both considering all the genes of the array or restricting analysis to those over-expressed by A17 cells (A17-signatures) (c). In all cases, basal-like samples clusterized more closely to breast than mesenchymal samples. (9.18 MB TIF) [file pone.0014131.s009.tif]
